# Targeting the Cascade Amplification of Macrophage Colony-stimulating Factor to Alleviate the Immunosuppressive Effects Following Radiotherapy

**DOI:** 10.34133/research.0450

**Published:** 2024-08-20

**Authors:** Zhiyun Liao, Yijun Wang, Yuxin Yang, Xixi Liu, Xiao Yang, Yu Tian, Suke Deng, Yan Hu, Jingshu Meng, Jie Li, Yue Deng, Zhiyuan Zhou, Wenwen Wei, Michelle Swift, Chao Wan, Yajie Sun, Kunyu Yang

**Affiliations:** ^1^ Cancer Center, Union Hospital, Tongji Medical College, Huazhong University of Science and Technology, Wuhan 430022, China.; ^2^ Institute of Radiation Oncology, Union Hospital, Tongji Medical College, Huazhong University of Science and Technology, Wuhan 430022, China.; ^3^ Hubei Key Laboratory of Precision Radiation Oncology, Wuhan, China.; ^4^ Department of Biochemistry and Molecular Medicine, University of Southern California, Los Angeles, CA 90089, USA.; ^5^ Department of Radiation Oncology, Dana-Farber Cancer Institute, Boston, MA 02215, USA.

## Abstract

Radiotherapy (RT) serves as the primary treatment for solid tumors. Its potential to incite an immune response against tumors both locally and distally profoundly impacts clinical outcomes. However, RT may also promote the accumulation of immunosuppressive cytokines and immunosuppressive cells, greatly impeding the activation of antitumor immune responses and substantially limiting the effectiveness of RT. Therefore, regulating post-RT immunosuppression to steer the immune milieu toward heightened activation potentially enhances RT’s therapeutic potential. Cytokines, potent orchestrators of diverse cellular responses, play a pivotal role in regulating this immunosuppressive response. Identifying and promptly neutralizing early released immunosuppressive cytokines are a crucial development in augmenting RT’s immunomodulatory effects. To this end, we conducted a screen of immunosuppressive cytokines following RT and identified macrophage colony-stimulating factor (MCSF) as an early up-regulated and persistent immune suppressor. Single-cell sequencing revealed that the main source of up-regulated MCSF derived from tumor cells. Mechanistic exploration revealed that irradiation-dependent phosphorylation of the p65 protein facilitated its binding to the MCSF gene promoter, enhancing transcription. Knockdown and chemical inhibitor experiments conclusively demonstrated that suppressing tumor cell-derived MCSF amplifies RT’s immune-activating effects, with optimal results achieved by early MCSF blockade after irradiation. Additionally, we validated that MCSF acted on macrophages, inducing the secretion of a large number of inhibitory cytokines. In summary, we propose a novel approach to enhance the immune activation effects of RT by blocking the MCSF-CSF1R signaling pathway early after irradiation.

## Introduction

Radiotherapy (RT) is a vital therapeutic approach in the fight against cancer, with approximately 50% to 70% of all patients undergoing this treatment [1,2]. Recent advances in cancer therapeutics underscore the pivotal role of activating the immune response in determining the overarching clinical efficacy of RT, which has been shown to influence both localized and distant malignancies, in murine models and potentially in humans [3]. While RT can provoke immune activation, and resulting in an “in situ vaccination”, its impact on the tumor microenvironment (TME) and downstream mechanisms are still poorly understood [4,5]. Notably, RT has the potential to accumulate immunosuppressive cytokines and cellular populations [6,7]. Therefore, to enhance the effectiveness of RT and improve patient survival, interventions aimed at targeting immunosuppressive factors, promoting immune activation, and shifting the immunologic balance toward heightened activation are essential in optimizing the post-irradiation immune environment.

Cytokines, relative small proteins secreted by immune and nonimmune cells, exert their biological influences predominantly via specific receptors, primarily orchestrating immune and inflammatory responses [8]. Within the intricate milieu of the TME, cytokines and their receptors assume multifaceted roles [9,10]. Cytokines demonstrate pleiotropy and redundancy in their actions, which depend on the receptors expressed by different cell types and the presence of additional signaling components [11]. Cytokines can interact with each other in ways that mutually influence their actions, leading to the formation of complex regulatory networks [12]. Moreover, the hierarchical cascade regulating cytokines is like a paradigm in cellular signal transduction, capable of inducing cascade amplification effects [8,13]. Early cytokine release serves as a crucial determinant in shaping inflammatory responses and constitutes a pivotal juncture in the orchestration of cytokine networks, rendering them promising targets for innovative therapeutic strategies [14].

RT has been demonstrated to promote the secretion of many immunosuppressive cytokines, including transforming growth factor-β (TGF-β), vascular endothelial growth factor (VEGF), and interleukin-6 (IL-6) [15–18]. Additionally, RT can elicit a response from inflammatory cytokines, thereby governing immune responses and tissue damage reactions [19,20]. Collectively, these cytokines sculpt the TME, impacting the immunogenicity of RT. However, the intricacies of cytokine cascade formation following RT remain poorly understood. Timely recognition of fluctuations in immunosuppressive cytokine levels following RT, coupled with timely intervention, may potently enhance the “in situ vaccine” effect of RT and deepen our understanding of the post-irradiation TME.

In this study, we aimed to identify the inhibitory cytokines activated by RT. To this end, we devised a multifaceted cytokine assay kit, able to evaluate alterations in cytokine profiles within Lewis lung cancer subcutaneous transplant tumors after 8 gray (Gy) irradiation. Among the array of cytokines evaluated, MCSF emerged as an early and sustained up-regulated candidate, warranting in-depth exploration. Additionally, we investigated the prospect of early-stage blockade of this cytokine to reactivate the immune microenvironment and antitumor immune activity following irradiation, thereby heightening the therapeutic efficacy of RT. Additionally, through the use of single-cell sequencing, we ascertained the primary cellular origins of MCSF after RT. Furthermore, we embarked upon a quest to unravel the underlying mechanism of its induction and delineate the specific cellular targets susceptible to its influence. This comprehensive investigation sheds light on the mechanisms of MCSF secretion, target cell specificity, and the potential therapeutic benefits of leveraging the up-regulation of this immunosuppressive cytokine following RT.

## Results

### Rapid escalation of MCSF following irradiation phase suppresses the sensitivity of tumors to RT

**Fig. 1.**
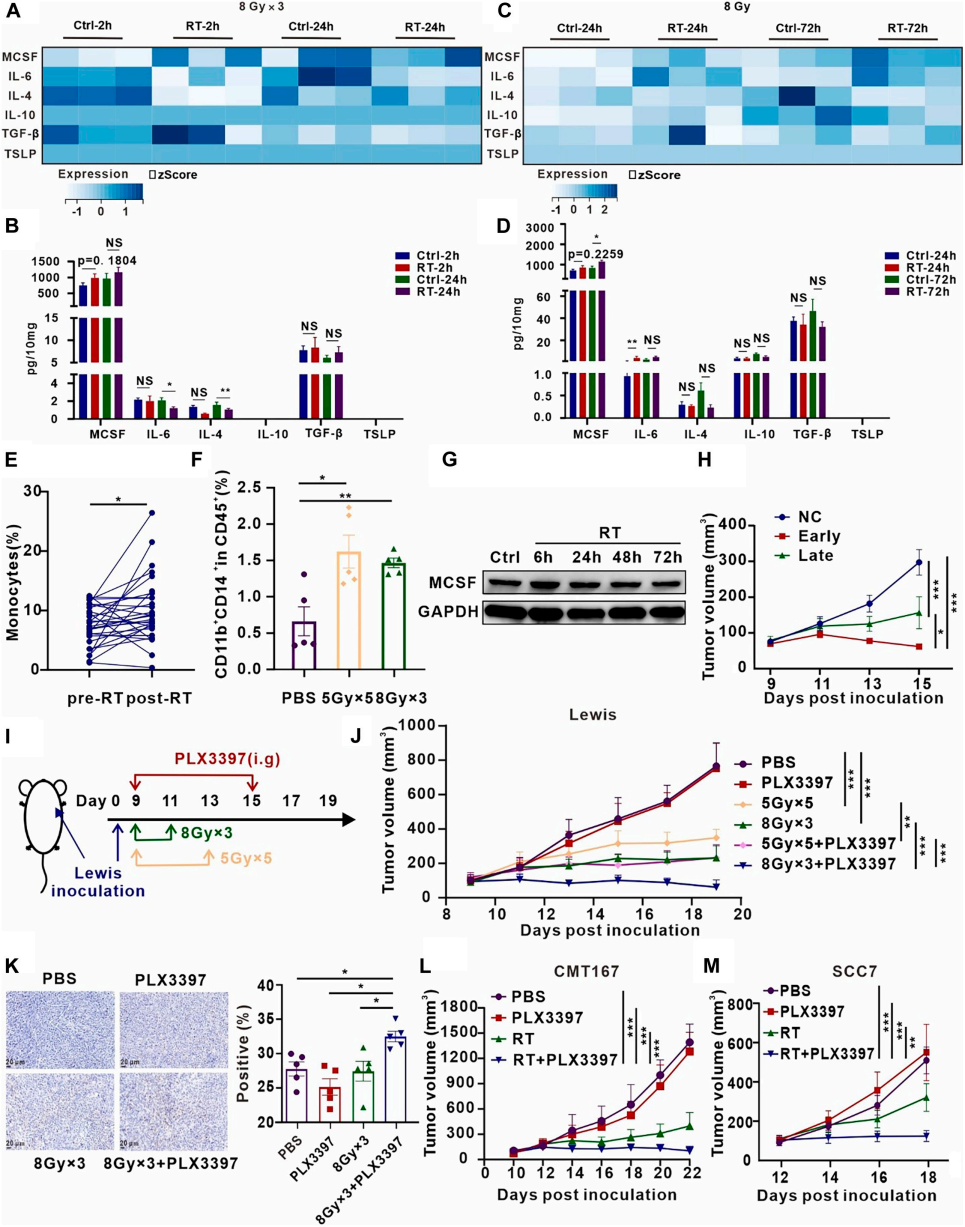
Inhibition of MCSF signaling after irradiation substantially attenuates tumor growth. (A) Heatmap illustrating alterations in cytokine expression profiles within Lewis subcutaneous transplant tumors at 2 and 24 h following 8 Gy ×3 irradiation. (B) Statistical presentation of differential cytokine expression patterns in subcutaneous tumors (*n* = 4 to 6 per group). (C) Heatmap depicting changes in cytokine expression profiles within Lewis subcutaneous transplant tumors at 24 and 72 h after a single 8-Gy irradiation. (D) Statistical representation of differential cytokine expression profiles within subcutaneous tumors (*n* = 4 to 6 per group). (E) Elevation in the proportion of monocytes in peripheral blood observed in patients with non-small cell lung cancer following RT (*n* = 30). (F) Flow cytometry analysis presenting variations in monocytes (CD11b^+^CD14^+^) within the peripheral blood of the Lewis subcutaneous transplant tumor model in response to the designated treatment regimens (*n* = 5 per group). (G) Protein expression levels of MCSF in Lewis cells after irradiation at 6, 24, 48, and 72 h were analyzed by Western blot (*n* = 3). (H) Tumor growth curves of the Lewis subcutaneous transplant tumor model subjected to different treatments (*n* = 7 per group). (I) Schematic representation of the treatment regimen involving RT in combination with the CSF1R inhibitor PLX3397. (J) Tumor growth curves of the Lewis subcutaneous transplant tumor model across different treatment groups (*n* = 7 per group). (K) Representative TUNEL immunohistochemical staining images of Lewis subcutaneous transplant tumors in the respective treatment groups (left). Quantification of the percentage of TUNEL staining-positive area, derived from 5 randomly selected fields within 3 replicate samples for each group (right) (*n* = 5 per group). (L) Tumor growth dynamics in the CMT167 subcutaneous transplant tumor model in response to the different treatments (*n* = 7 per group). (M) Tumor growth dynamics in the SCC7 subcutaneous transplant tumor model in response to the different treatments (*n* = 9 per group). *P < 0.05; **P < 0.01; ***P < 0.001.

To explore potential alterations in inhibitory cytokine secretion following irradiation, we devised a versatile multi-cytokine assay with the capability to detect 6 inhibitory cytokines: MCSF, IL-6, IL-4, IL-10, TGF-β, and thymic stromal lymphopoietin (TSLP). Utilizing this assay, we evaluated tumor specimens obtained from a Lewis subcutaneous transplant tumor model at 2 and 24 h following exposure to either sham irradiation or 3 consecutive days of 8-Gy irradiation. While both MCSF and TGF-β displayed an upward trend, the degree of up-regulation was not sufficiently pronounced (Fig. 1A and B). Subsequently, we harvested tumor samples at 24 and 72 h after a single 8-Gy irradiation, revealing a substantial and statistically significant elevation in MCSF levels at 72 h (Fig. 1C and D). MCSF exhibited a notably higher baseline expression in comparison to other inhibitory cytokines (Fig. 1C and D). These findings collectively affirm that the inhibitory cytokine MCSF undergoes rapid and sustained elevation after irradiation. In vitro supplementation experiments underscored that MCSF induced heightened transcription levels of inhibitory cytokines, including IL-4, VEGF, and TGF-β (Fig. S1). MCSF is recognized as an important factor in the differentiation of myeloid progenitor cells into various cellular lineages, encompassing monocytes, macrophages, dendritic cells (DCs), and osteoclasts [21]. Within the TME, MCSF up-regulation stimulates macrophages toward the M2 phenotype and the secretion of immunosuppressive cytokines, such as TGF-β and IL-10, thus inhibiting antitumor immune responses [22]. Additional scrutiny uncovered a significant increase in the proportion of monocytes in peripheral blood following RT in 30 lung cancer patients (Fig. 1E). Similarly, in the Lewis tumor transplantation model, we noted an increase in monocyte proportions in peripheral blood following 2 different RT treatments (Fig. 1F). Therefore, we chose to concentrate our investigation on MCSF as the primary cytokine of interest for subsequent studies.

Disruption of the MCSF signaling pathway for clinical applications primarily targets colony-stimulating factor 1 receptor (CSF1R) [23,24]. In our study, we harnessed the CSF1R inhibitor PLX3397 in combination with RT. In vitro experiments revealed that the protein expression of MCSF exhibited the most significant elevation at 6 h after radiation (Fig. 1G). Additionally, a comparative analysis between the administration of PLX3397 therapy at the initiation of RT and its administration 48 h following RT showed that early inhibition of CSF1R signaling augmented the efficacy of RT in restraining tumor growth (Fig. 1H). Indeed, early blockade of the CSF1R-MCSF pathway combined with RT significantly inhibited tumor growth compared to phosphate-buffered saline (PBS), PLX3397 only, and RT only (Fig. 1I and J and Fig. S2A to C). The 8 Gy ×3 regimen combined with PLX3397 showed better therapeutic efficacy compared to the 5 Gy ×5 regimen (Fig. 1J). Terminal deoxynucleotidyl transferase-mediated deoxyuridine triphosphate nick end labeling (TUNEL) assays demonstrated a notably higher proportion of tumor cells undergoing apoptosis in the RT combined with PLX3397 group as opposed to other groups (Fig. 1K). Similar inhibitory effects on tumor growth were observed in the subcutaneous model of CMT167 lung cancer and SCC7 head and neck squamous cell carcinoma in mice (Fig. 1L and M and Fig. S2D and E). Furthermore, the combination therapy exhibited favorable safety profiles in mice (Fig. S3). The aforementioned findings suggest that combining RT with a CSF1R inhibitor has shown effectiveness in impeding tumor growth in murine models of Lewis lung carcinoma, CMT167 lung carcinoma, and SCC7 head and neck squamous cell carcinoma in subcutaneous transplant tumor models.

### Early blockade of the MCSF pathway promotes T cell activation

**Fig. 2.**
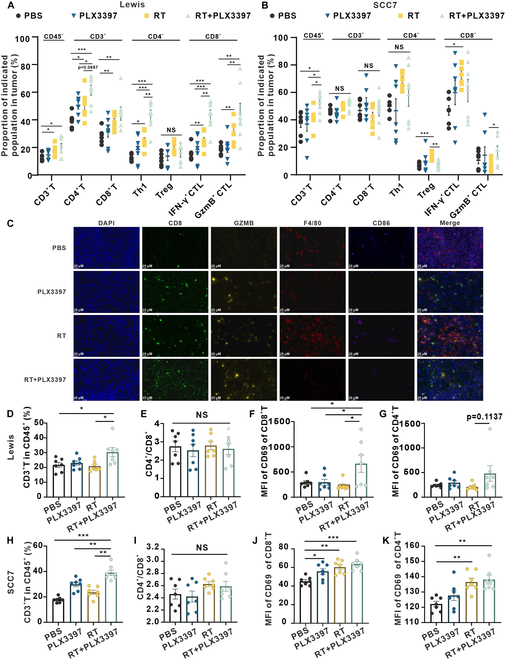
Early blocking of MCSF pathway activates T cell-mediated immunity. (A) Flow cytometry analysis depicting alterations in T cell populations within the Lewis subcutaneous tumor across various treatment groups (*n* = 7 per group). (B) Flow cytometry analysis illustrating changes in T cell populations within the SCC7 subcutaneous tumor in different groups (*n* = 7 per group). (C) Immunofluorescence staining of tumor tissue displaying CD8 (green) and granzyme B (yellow), as well as F4/80 (red) and CD86 (purple) expression following diverse treatments. Scale bars, 20 μm. (D to G) Flow cytometry analysis presenting variations in T cell populations within the spleen of the Lewis subcutaneous transplant tumor model in response to the different treatments (*n* = 7 per group). (H to K) Flow cytometry analysis presenting variations in T cell populations within the spleen of the SCC7 subcutaneous transplant tumor model in response to the different treatments (*n* = 7 per group).

We next performed an in-depth exploration of the mechanisms underlying the inhibition of tumor growth upon MCSF pathway blockade, including multi-cytokine analysis of tumor tissues collected from 4 distinct groups. The analysis revealed that inhibiting the MCSF signal after irradiation did not impact the secretion of local inhibitory cytokines (Fig. S4). To unravel the antitumor mechanism of combining RT with the CSF1R inhibitor, flow cytometry analysis was conducted on immune cell subsets within the subcutaneous tumor tissues of Lewis lung carcinoma and SCC7 head and neck squamous cell carcinoma models. Following the gating strategy shown in Fig. S5, we analyzed changes in T cell populations. Notably, the proportion of CD3^+^ T cells was significantly increased in the combination treatment and RT only group when compared to the PBS groups (Fig. 2A). Within the combination treatment group, a marked elevation was observed in the proportion of T helper 1 (T_H_1) cells among CD4^+^ lymphocytes, as well as cytotoxic T lymphocytes (CTLs) secreting interferon-γ (IFN-γ) among CD8^+^ lymphocytes, as compared to the PBS and RT only groups (Fig. 2A). In the context of SCC7 head and neck squamous cell carcinoma subcutaneous tumor tissue, the combination treatment group also exhibited a significantly increased proportion of CD3^+^ T cells in comparison to the PBS, PLX3397 only, and RT only groups (Fig. 2B). Moreover, in contrast to the PBS group, the RT group showed a noteworthy increase in the proportion of regulatory T cells (T_regs_) within the CD4^+^ T lymphocyte population, whereas the combination treatment group displayed a significant reduction in T_regs_ (Fig. 2B). Additionally, the combination treatment group displayed a substantially higher proportion of CTLs secreting granzyme B (GzmB) within the CD8^+^ T lymphocyte subset when compared with the RT group, as depicted in Fig. 2B. Immunofluorescence experiments validated these alterations in T cell subpopulations and discerned noteworthy changes in myeloid cells as well (Fig. 2C).

We proceeded to explore whether the impact of RT combined with PLX3397 extended beyond the TME to influence systemic T cell immunity. Flow cytometry analysis was performed on spleen tissue harvested from both the Lewis and SCC7 subcutaneous transplant tumor models. In the Lewis subcutaneous transplant tumor model, the combination treatment group exhibited a significant increase in the proportion of CD3^+^ T cells compared to the PBS and RT only groups (Fig. 2D). No discernible differences in the proportions of CD4^+^ and CD8^+^ T cells were noted among the groups (Fig. 2E). Notably, CD69 expression, an activation marker for CD8^+^ T lymphocytes, significantly increased in the combination treatment group in contrast to the PBS group, PLX3397 group, and RT group within the Lewis subcutaneous transplant tumor model (Fig. 2F). A trend toward heightened CD69 expression on CD4^+^ T lymphocytes was also observed in the Lewis model (Fig. 2G). Within the SCC7 subcutaneous transplant tumor model, the combination treatment group displayed a substantial increase in the proportion of CD3^+^ T cells when compared with the other groups (Fig. 2H), with no significant differences detected in the proportions of CD4^+^ and CD8^+^ T cells among the groups (Fig. 2I). The combination treatment group displayed an increase in the CD69 expression on CD4^+^ and CD8^+^ T cells, compared with the PBS group (Fig. 2J and K).

Our findings collectively highlight that blocking the MCSF pathway not only enhances the quantity and function of T_H_1 and CTLs within the TME but also enhances the activation of T cells in the spleen and amplifies the systemic antitumor immune responses.

### Early blockade of the MCSF pathway alleviates the myeloid-derived suppressor factors

**Fig. 3.**
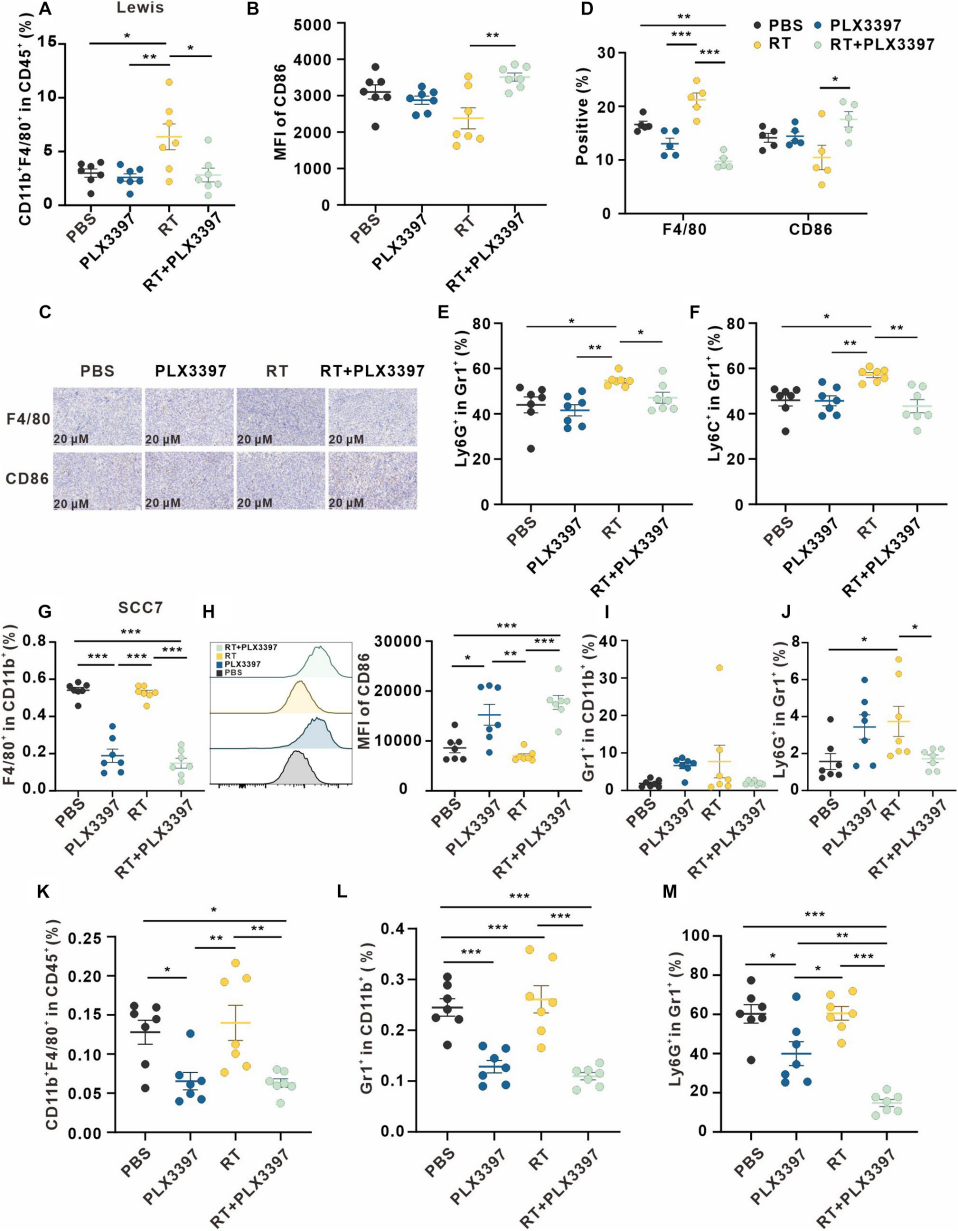
Early blockade of the MCSF pathway promotes macrophage polarization toward M1 phenotype and reduces MDSCs. (A and B) Flow cytometry analysis depicting alterations in macrophages within the Lewis subcutaneous tumor across various treatment groups (*n* = 7 per group). (C) Representative immunohistochemical staining of Lewis subcutaneous tumors in corresponding groups. (D) Five fields were randomly selected from 3 replicates for each group, and the percentage of staining positive area was counted. (E and F) MDSCs in Lewis subcutaneous tumors in corresponding treatment groups were analyzed using flow cytometry (*n* = 7 per group). (G to J) Myeloid cells in SCC7 subcutaneous tumor in corresponding treatment groups (*n* = 7 per group) were analyzed using flow cytometry. (K to M) Myeloid cells in the spleen of SCC7 subcutaneous transplant tumor model in corresponding treatment (*n* = 7 per group) were analyzed using flow cytometry.

Building on our previous immunofluorescence experiments, we conducted flow cytometric analysis to evaluate myeloid cell populations within Lewis subcutaneous transplant tumors (Fig. S6A). Intriguingly, in comparison to the PBS group, the RT group exhibited a significant increase in the proportion of macrophages among the CD45^+^ immune cell population (Fig. 3A). In contrast, the combination treatment of PLX3397 and RT elicited a significant reduction in macrophages (Fig. 3A). We next evaluated CD86 expression on macrophages, a marker of classical M1 macrophages. We observed a marked increase in CD86 expression upon combination treatment group, indicating a potential transition toward a phenotypic reprogramming characterized by classical M1 macrophage polarization (Fig. 3B to D). Furthermore, there was no significant difference observed in CD206 expression in macrophages between the groups (Fig. S6B). Next, our aim was to characterize myeloid-derived suppressor cells (MDSCs), a group known for their multifaceted orchestration of immune suppression through a complex array of pathways. These cells play a crucial role in inducing T cell dysfunction through various mechanisms, including up-regulation of PD-L1, inhibition of T cell proliferation, and activation by depleting arginine, hindering antigen presentation by DCs, and releasing immunosuppressive factors like IL-10 and TGF-β, ultimately promoting the expansion of T_regs_ [25–28]. Compared to the PBS group, the RT group showed a significant increase in the proportion of polymorphonuclear (PMN)-MDSCs (Gr1^+^Ly6G^+^Ly6C^-^) and M-MDSCs (Gr1^+^Ly6G^-^Ly6C^+^) within MDSCs (CD11b^+^Gr1^+^), while the combination of PLX3397 significantly reduced the proportion of both subsets (Fig. 3E and F).

Subsequently, in the context of SCC7 subcutaneous tumors, we observed similar results. Evidently, both PLX3397 and the combination treatment significantly increased macrophage abundance within the TME (Fig. 3G). Importantly, the combination treatment substantially increased the CD86 expression of the macrophages, suggestive of an increase in M1 polarization (Fig. 3H). In addition, compared to the PBS group, the proportion of MDSCs in the RT group increased, while the combination of PLX3397 with RT reduced the proportion of MDSCs (Fig. 3I). In the RT group, the proportion of PMN-MDSCs in MDSCs was significantly increased compared to the PBS group, while the combination treatment with PLX3397 significantly reduced the proportion of PMN-MDSCs in MDSCs (Fig. 3J and Fig. S6C). Intriguingly, in spleen tissues derived from SCC7 subcutaneous transplant tumor model, the combination treatment resulted in a significant reduction in macrophages within the CD45^+^ immune cell population (Fig. 3K). The combination treatment led to a significant reduction in the proportion of MDSCs in spleen tissues compared to RT only and PBS groups (Fig. 3L). Additionally, there was a significant decrease in the proportion of PMN-MDSCs in spleen tissues compared to all other groups (Fig. 3M). Besides T cells and myeloid cells, we evaluated DCs, NK cells, and B cells in the tumor tissues of Lewis subcutaneous transplant tumor model, finding no differences between the combined treatment and RT only groups (Fig. S7).

These results indicate that blocking the MCSF pathway promotes M1 polarization of macrophages and reduces MDSC infiltration in the TME, as well as decreases the proportion of macrophages and MDSCs in the spleen, indicating an alleviation in the myeloid-suppressive TME.

### Single-cell sequencing unveils tumor cells as the primary source of MCSF in the post-irradiation microenvironment

**Fig. 4.**
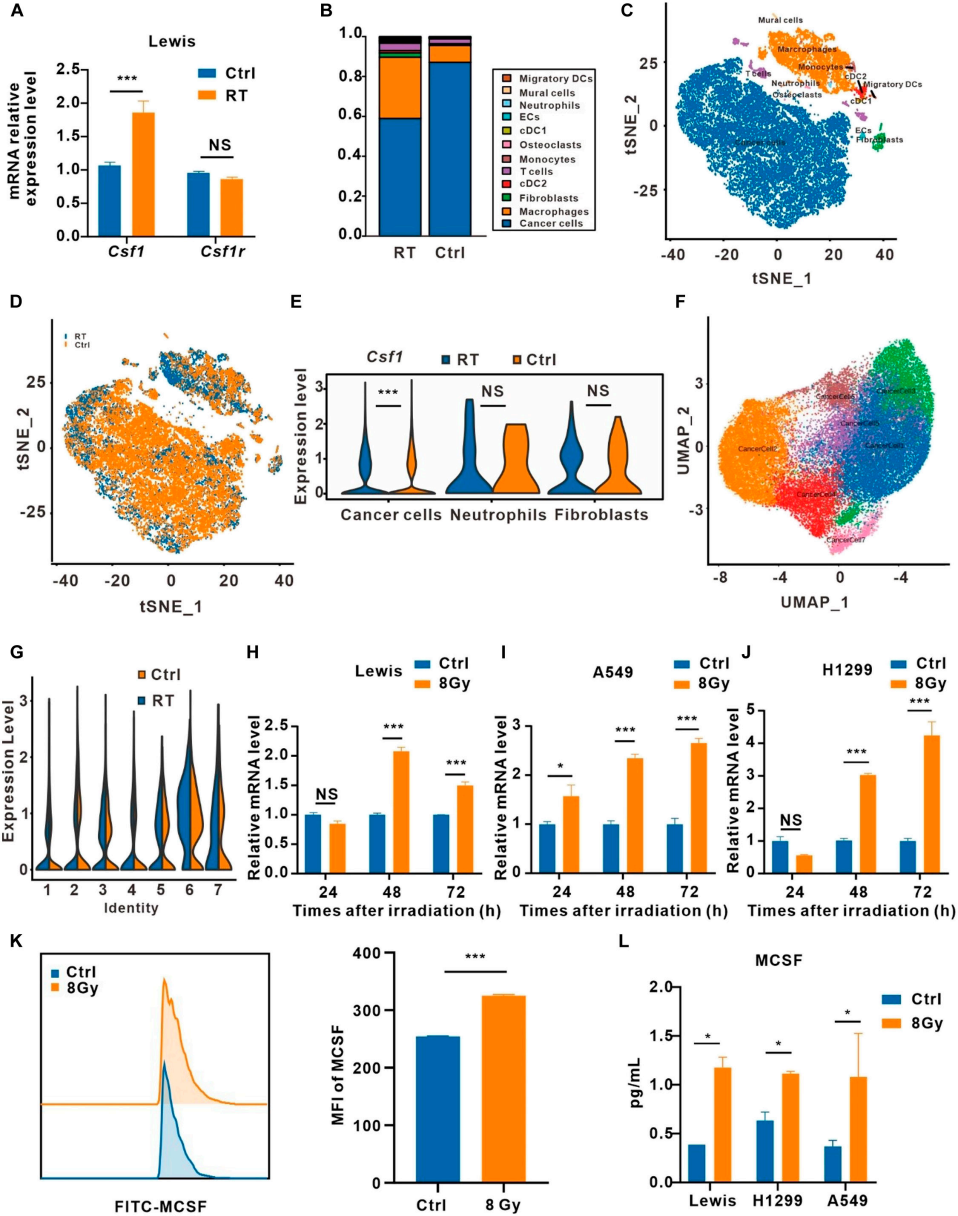
MCSF elevated after irradiation mainly came from tumor cells. (A) Analysis of *Csf1* and *Csf1r* mRNA expression in Lewis subcutaneous transplant tumors by RT-qPCR (*n* = 3). (B) Histograms of single sample cell compositions. (C and D) t distributed stochastic neighbor embedding (tSNE) representation for unirradiated (Ctrl) and irradiated (RT) tumor tissue. Color-coded for cell type. (E) Comparison of *Csf1* expression in 3 major clusters of cells. (F) Uniform manifold approximation and projection (UMAP) representation for tumor cell clusters. (G) Comparison of *Csf1* expression in 7 tumor cell clusters. (H to J) *Csf1* mRNA expression levels in Lewis (H), A549 (I) and H1299 (J) cells after irradiation (*n* = 3). (K) Flow cytometry analysis of MCSF expression in Lewis cells after irradiation. MFI, mean fluorescence intensity (*n* = 3). (L) Cell supernatants from irradiated Lewis, A549, and H1299 cells were analyzed by ELISA for MCSF (*n* = 3). *P < 0.05; **P < 0.01; ***P < 0.001.

To further determine the mechanisms underlying the increased MCSF levels after irradiation, we conducted real-time quantitative polymerase chain reaction (RT-qPCR) analysis on Lewis subcutaneous transplant tumors following exposure to 8-Gy irradiation. We observed a significant increase in transcription levels of the MCSF gene (CSF1) after irradiation in comparison to the control group (Fig. 4A). Interestingly, there was no observable shift in its receptor, CSF1R (Fig. 4A). GEXSCOPE single-cell transcriptome analysis revealed alterations in the cellular landscape. Irradiated tumor tissues exhibited increased proportions of macrophages, fibroblasts, T cells, and cDC2, accompanied by a reduction in the proportion of tumor cells when compared with their control counterparts (Fig. 4B to D and Fig. S8A and B). Remarkably, the up-regulation of MCSF expression was specifically observed in tumor cells following irradiation, whereas neutrophils and fibroblasts remained unaffected (Fig. 4E). Cluster analysis further dissected the tumor cell population into 7 distinct subgroups, with notable increments in cells within subgroups 2, 3, and 5 after irradiation (Fig. 4F and Fig. S8C). Moreover, gene expression profiles exhibited marked shifts among these tumor cell subgroups after irradiation (Fig. S8D). Notably, MCSF expression in subgroups 3 and 6 of tumor cells witnessed a substantial increase after irradiation (Fig. 4G), pinpointing tumor cells as the primary source of elevated MCSF levels following irradiation.

RT-qPCR analysis confirmed the results of single-cell transcriptome analysis, revealing a significant up-regulation of MCSF expression in Lewis, A549, and H1299 cells after irradiation (Fig. 4H and J). Flow cytometry analysis further validated these findings, demonstrating an elevation in MCSF expression within tumor cells after irradiation (Fig. 4K). Furthermore, enzyme-linked immunosorbent assay (ELISA) assays performed from the supernatant of irradiated tumor cells confirmed an increase in the secretion of MCSF (Fig. 4L), suggesting that the heightened MCSF levels after irradiation primarily derived from tumor cells.

### Irradiation-induced activation of the NF-κB signaling pathway in tumor cells leads to up-regulation of MCSF

**Fig. 5.**
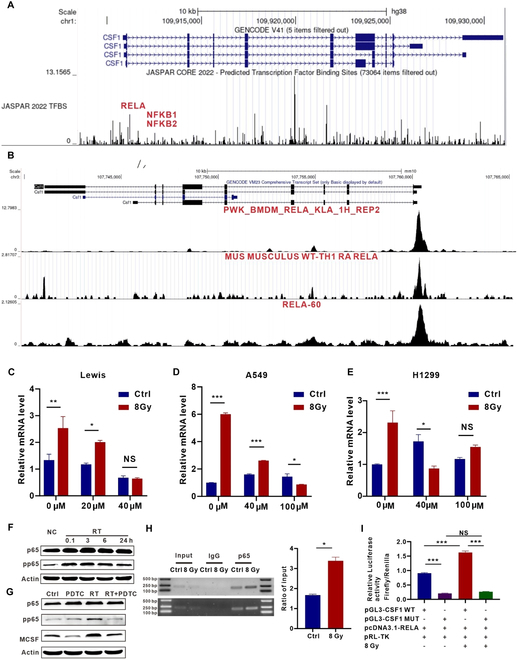
P65 binding to the MCSF gene promoter is enhanced after irradiation. (A) Transcription factors for MCSF (gene name: *Csf1*) were predicted using the JASPAR database. (B) ChIP-seq data of murine cell lines predicted p65 (RELA) binding to the *Csf1*. (C to E) Analysis of *Csf1* mRNA expression in Lewis (C), A549 (D), and H1299 (E) cells treated with PDTC after irradiation (*n* = 3). (F) Protein expression levels of p65 and pp65 in H1299 cells after irradiation at 0.1, 3, 6, and 24 h were analyzed by Western blot (*n* = 3). (G) Protein expression levels of p65, pp65, MCSF in H1299 cells treated by PDTC after irradiation were analyzed by Western blot (*n* = 3). (H) In unirradiated and irradiated H1299 cells (*n* = 3), ChIP assays were performed. Left panel shows representative gel electrophoresis images. Right panel shows p65 levels in the gene promoter region normalized to input. (I) Relative luciferase activity was evaluated in different groups. WT, wild type; MUT, mutation; pRL-TK, renilla luciferase plasmid (*n* = 3). *P < 0.05; **P < 0.01; ***P < 0.001.

To unravel the underlying mechanism driving irradiation-induced tumor cell secretion of MCSF, we used a transcription factor prediction analysis, revealing nuclear factor κB (NF-κB) as a potential regulator of MCSF expression (Fig. 5A and B). NF-κB, an essential transcription factor in the inflammatory response, governs an array of genes [29,30]. To determine the role of NF-κB in regulating MCSF expression, we utilized a well-established NF-κB inhibitor, pyrrolidinedithiocarbamate ammonium (PDTC), which primarily impedes the nuclear translocation of NF-κB [31,32]. Treatment with PDTC effectively reduced the irradiation-induced increase in MCSF expression in Lewis, A549, and H1299 cells (Fig. 5C to E). Furthermore, Western blot analysis confirmed the activation of the NF-κB signaling pathway after irradiation (Fig. 5F). PDTC intervention effectively attenuated the phosphorylation of p65 and the concomitant up-regulation of MCSF expression following irradiation (Fig. 5G), conclusively demonstrating that the NF-κB signaling pathway exerts regulatory control over MCSF expression following irradiation.

To ascertain whether NF-κB directly regulates the expression of the MCSF gene in response to irradiation, we conducted chromatin immunoprecipitation (ChIP)-qPCR experiments. We observed enhanced binding of p65 to the MCSF gene promoter after irradiation in H1299 cells (Fig. 5H). This binding demonstrated a 2.03-fold increase compared to the control group (Fig. 5H). Similar findings were also observed in Lewis cells (Fig. S9). The dual luciferase reporter assay further confirmed enhanced binding of p65 to the MCSF gene promoter after irradiation (Fig. 5I). Collectively, these results suggest that irradiation-induced up-regulation of MCSF is orchestrated through the NF-κB signaling pathway.

### Reducing tumor cell-derived MCSF after irradiation alters the cytokine profile of TME

**Fig. 6.**
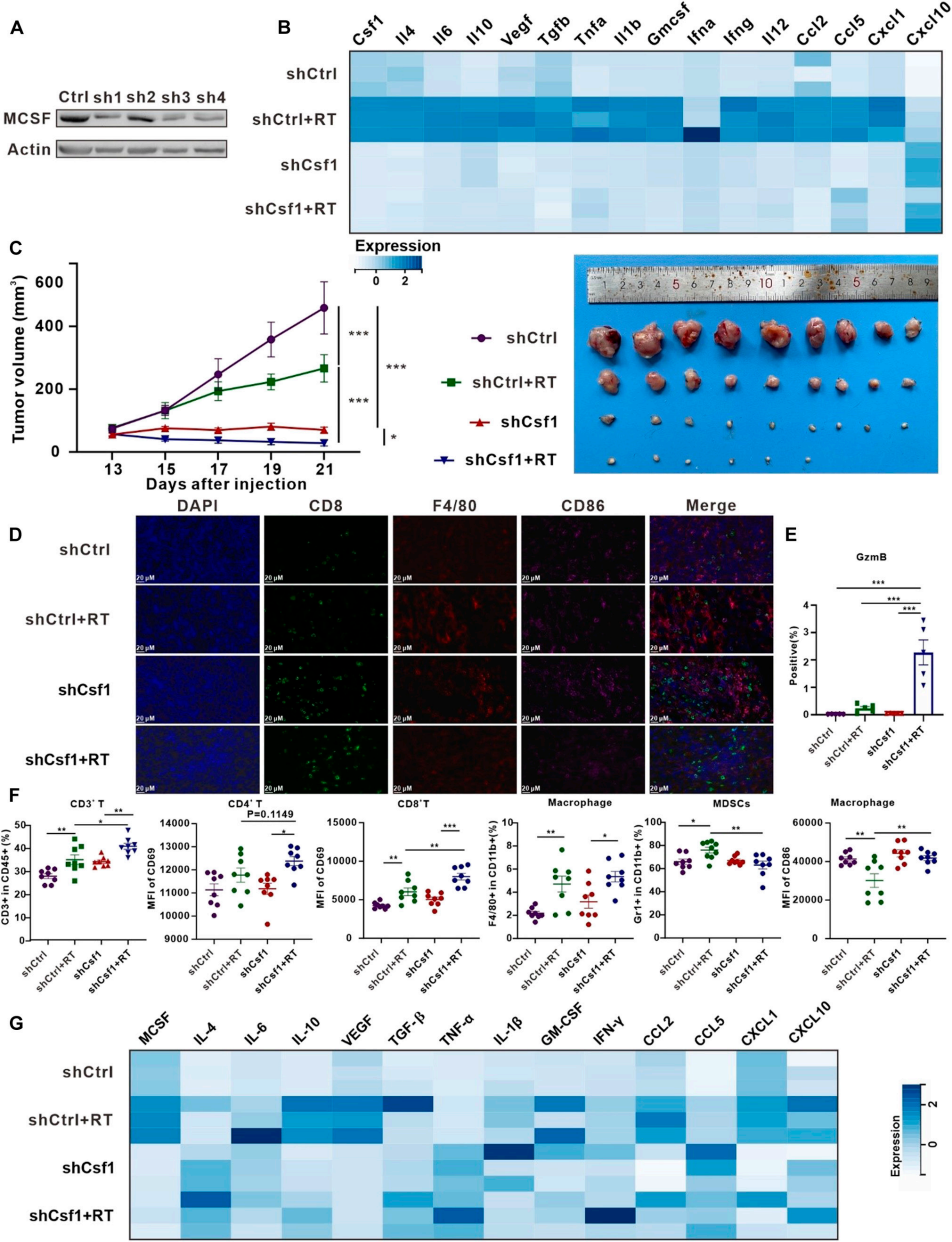
Reducing tumor cell-derived MCSF after irradiation diminishes the cascading amplification of immunoinhibitory cytokines. (A) Lewis cells were transfected with either control or shCsf1 lentivirus, and subsequently, samples were collected for Western blot analysis to assess the expression levels of MCSF (*n* = 3). (B) RT-qPCR analysis of the expression levels of cytokines in Lewis stable cells (shCtrl and shCsf1) after irradiation (*n* = 3). (C) Growth curves of tumors in the Lewis subcutaneous transplant tumor model treated in corresponding treatment groups (left). Visual representation of tumors in Lewis subcutaneous transplant tumor model treated by corresponding treatment groups (right) (*n* = 9 per group). (D) Immunofluorescence staining of CD8 (green), F4/80 (red), and CD86 (purple) expression on the tumor tissue after different treatments. Scale bars, 20 μm. (E) Five fields were randomly selected from 3 replicates for each group, and the percentage of staining positive area of GzmB was counted. (F) Flow cytometry analysis of T cells, macrophages, and myeloid cells in Lewis subcutaneous tumors. (G) Differences in the expression of different cytokines in Lewis subcutaneous transplant tumor models according to their treatment (*n* = 3).

Given that tumor cells are the primary source of MCSF in the TME following RT, we depleted Csf1 of Lewis cells using short hairpin RNAs (shRNAs) (Fig. 6A). Notably, the loss of Csf1 blocked the increase of immunoinhibitory cytokines in vitro (Fig. 6B and Fig. S10A). In the Lewis subcutaneous transplant tumor model, we observed remarkable inhibition of tumor growth within the shCsf1 group. Furthermore, following RT, there was a further reduction in tumor volume, with 3 cases even achieving complete remission (Fig. 6C). Immunofluorescence analysis of tumor tissues unveiled the increase in the proportion of T cells in the shCsf1 + RT group (Fig. 6D). The expression of GzmB in the shCsf1 + RT group was up-regulated, indicating the activation of T cells (Fig. 6E and Fig. S10B). Flow cytometry analysis of splenic tissues showed that the shCsf1 + RT group had a higher percentage of CD3^+^ T cells, along with a higher percentage of CD4^+^ and CD8^+^ T cells expressing the activation marker CD69 (Fig. 6F). Additionally, this group showed a lower percentage of MDSCs and macrophages polarized toward M1 phenotypes (Fig. 6F). Intriguingly, multifactorial analysis of tumor tissues unveiled a significant decrease in the expression of immunosuppressive cytokines, encompassing MCSF, IL-10, and VEGF, within the shCsf1 + RT group (Fig. 6G and Fig. S10C). Conversely, there was a noticeable surge in the expression of pro-inflammatory cytokines such as TNF-α and IL-1β (Fig. 6G and Fig. S10C). These compelling findings suggest that the reduction of tumor-derived MCSF can locally and systemically trigger antitumor immune responses, orchestrate shifts in the cytokine expression profile, and propel a transition toward immune activation, thus restoring immune equilibrium after irradiation.

### MCSF regulates the function of macrophages and induces irradiation-mediated immunosuppression

**Fig. 7.**
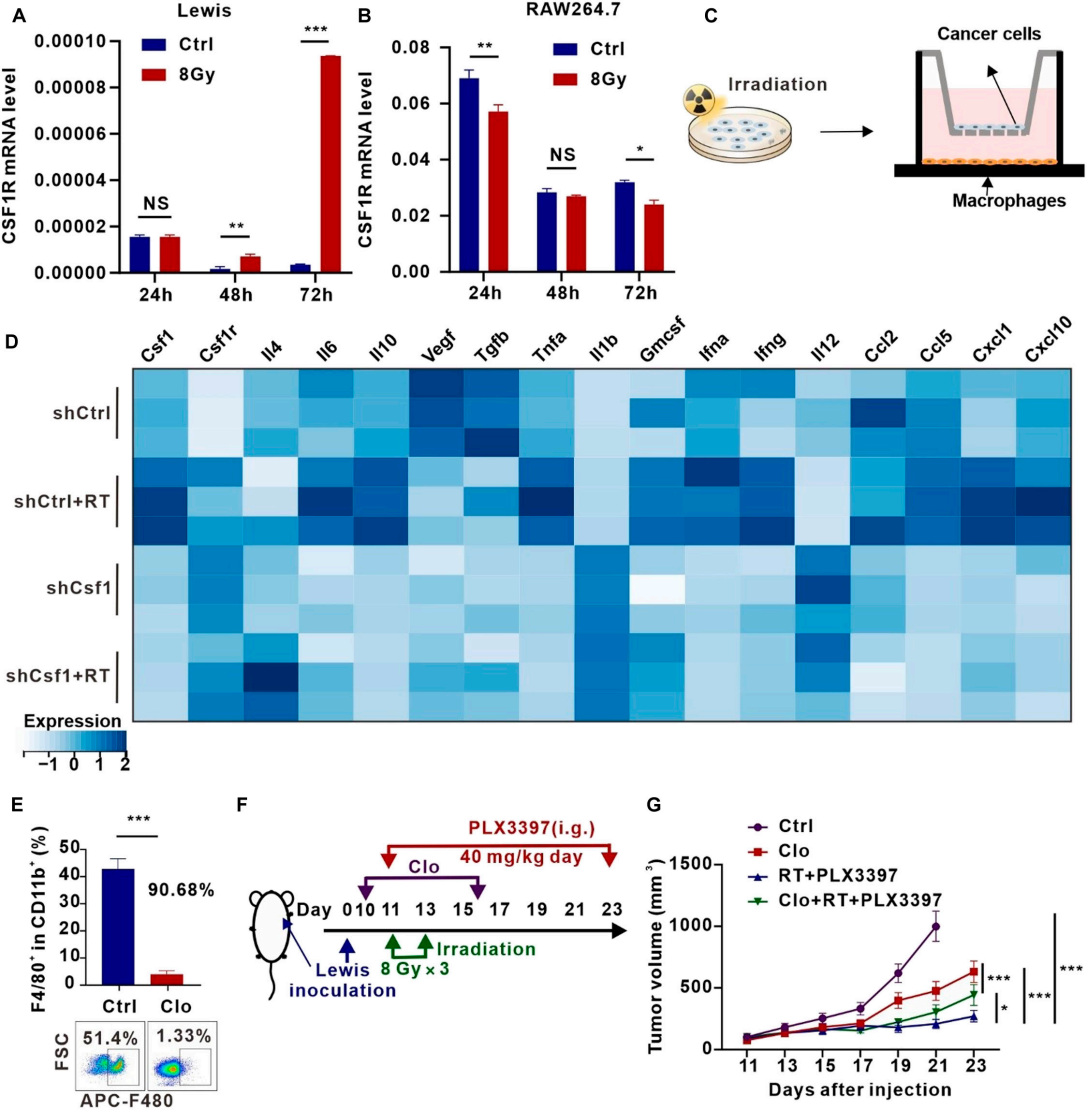
RT combined with the CSF1R inhibitor exerts tumor-suppressive effects partly by acting on macrophages. (A and B) *Csf1r* mRNA expression levels in Lewis (A) and RAW264.7 (B) after irradiation for 24, 48, and 72 h (*n* = 3). (C) Schematic illustration of the coculture. (D) In BMDMs cocultured with Lewis stable cells (shCtrl and shCSF1), RT-qPCR was used to identify cytokines expressed in the cells (*n* = 3). (E) Flow cytometry analysis of clearance efficiency of clodronate liposomes (Clo) on macrophages in peripheral blood (*n* = 3). (F) Schema illustrating the treatment plan of RT combined with the CSF1R inhibitor PLX3397 and Clo. (G) Growth curves of tumors in the Lewis subcutaneous transplant tumor model in the different groups (*n* = 7 per group). *P < 0.05; **P < 0.01; ***P < 0.001.

MCSF exerts its effects by binding to its receptor CSF1R, which is mainly expressed on macrophages, DCs, osteoclasts, and other cells [21]. RT-qPCR analysis showed low absolute expression levels of CSF1R in tumor cells but high expression levels in the macrophage cell line RAW264.7 (Fig. 7A and B), indicating that macrophages might be the main target of irradiated tumor cell-derived MCSF. Studies have shown that macrophages can secrete a variety of cytokines to regulate the function of immune cells in the TME [33,34]. To determine whether tumor cell-derived MCSF can influence cytokine secretion from macrophages, we conducted coculture experiments (Fig. 7C). Coculture with irradiated tumor cells significantly elevated the expression of immunosuppressive cytokines in mouse bone marrow-derived macrophages (BMDMs), including MCSF, IL-6, and IL-10, and elevated pro-inflammatory cytokines IL-1β, TNF-α, IFN-α, and IFN-γ, while reducing tumor cell-derived MCSF decreased the expression of MCSF, IL-6, IL-10, and up-regulated the expression of IL-1β and IL-12 (Fig. 7D and Fig. S11A). These results suggest that tumor cell-derived MCSF after irradiation alters the cytokine profile of macrophages, indicating that macrophages may be the target cells of MCSF.

Macrophage clearance experiments were conducted to confirm whether RT combined with PLX3397 acted through macrophages to inhibit tumor growth and improve the after irradiation immune microenvironment. Clodronate liposomes (Clo) can induce macrophage apoptosis after phagocytosis by releasing chlorophosphate, making it a useful tool for macrophage depletion [35,36]. Clo effectively cleared macrophages in the spleen and peripheral blood with a clearance efficiency of 92.2% in the spleen and 90.68% in the peripheral blood (Fig. 7E and Fig. S11B). Tumor growth curves revealed that RT combined with PLX3397 significantly restrained tumor growth in the absence of macrophage clearance. However, upon macrophage depletion, the tumor-suppressive effect of the combination treatment was noticeably attenuated but still exceeded that of macrophage depletion only (Fig. 7G). Taken together, tumor cell-derived MCSF acted on macrophages to alter the macrophage cytokine profile. RT combined with the CSF1R inhibitor exerted tumor-suppressive effect partly by acting on macrophages.

## Discussion

Insufficient immune activation by RT may hamper the sustained antitumor effects in vivo [37,38]. The effects of RT on the immune microenvironment could be transmitted through a large number of cytokines, such as TGF- β, IFN- γ, IL-1β, CCL2, and CXCL16, which accumulate in the post-irradiation TME [39–41]. In this study, we aimed to explore the root causes of suboptimal immune activation, delving into the cascade amplification of immunosuppressive cytokines triggered by RT. We discovered that p65 protein in irradiated tumor cells undergoes rapid phosphorylation, binding to the MCSF gene promoter to enhance its transcriptional expression, thus contributing to the secretion of MCSF into the TME. This MCSF subsequently impacts macrophages, prompting the release of an array of inhibitory cytokines. By reducing MCSF derived from tumor cells after irradiation, we effectively disrupted the cascade initiated by MCSF, thereby ameliorating the post-irradiation immune microenvironment. This intervention further enhanced the local and systemic immune-activating effects of RT, tilting the immune balance toward heightened immune activation.

Current research is centered on augmenting the immune response provoked by RT, potentially through inhibiting the tumor-intrinsic E3 ligase tumor cell-intrinsic tripartite motif-containing 21 (TRIM21), to further bolster antitumor immunity [42]. Despite ongoing research endeavors, the specific mechanisms impeding irradiation-induced antitumor immunity remain elusive. The cascade amplification of suppressive cytokines induced by irradiation may indeed play a pivotal role in impairing irradiation-induced antitumor immunity. In this study, we highlight the pivotal role of MCSF, an inhibitory cytokine, which is up-regulated during the early phases after irradiation. Remarkably, upon knocking down MCSF expression in tumor cells, 3 mice achieved complete remission after RT in the Lewis subcutaneous transplant tumor model (Fig. 6C). Furthermore, we found that MCSF is instrumental in initiating the cascade amplification of cytokines within the TME after irradiation, proposing an innovative avenue to enhance the immune activation effects of RT.

Existing studies have also explored the relationship between the inhibitory cytokine MCSF and tumor immunity. Senescent tumor cells, for instance, secrete MCSF to drive macrophage polarization toward the M2 subtype and inhibit CD8^+^ T cell activation [43]. Additionally, RT can stimulate the tyrosine kinase ABL1 in prostate cancer cells, leading to its translocation from the cytoplasm to the nucleus where it binds to the MCSF gene promoter, thus promoting MCSF secretion from prostate cancer cells [44]. Beyond tumor cells, various cell types such as neutrophils, fibroblasts, endothelial cells, stromal cells, macrophages, and smooth muscle cells have also been found to secrete MCSF, contributing to immune tolerance [45–47]. In this study, we elucidated that tumor cells represent a significant source of elevated MCSF following RT through single-cell transcriptome analysis of post-irradiation tumor tissues, thereby further enhancing our understanding of the immune landscape within the post-irradiation TME.

There are also some weaknesses in present study. The primary screening of the up-regulated immune inhibitory cytokines in the early post-irradiation period was limited in scope. Future studies will expand the profiles of immune inhibitory cytokines following irradiation. The effects of individual cytokines on tumor growth depend on the context and are influenced by synergistic interactions within the complex cytokine network [12]. The specific mechanism by which MCSF affects downstream cytokines is still not fully elucidated. In subsequent studies, the current inadequacies of the study will be further refined.

In summary, this study underscores the importance of targeting the cascade amplification of tumor cell-derived MCSF to mitigate immunosuppressive effects after RT, ultimately activating both innate and adaptive immunity. It holds the potential to overcome the bottleneck of RT ineffectiveness, and offers novel insights and methodologies for enhancing the post-irradiation immune microenvironment—a development with significant clinical applicability.

## Materials and Methods

### Chemical reagents

The following primary antibodies were utilized in the research: rabbit anti-NF-κB p65 (Cell Signaling Technology, #8242, 1:1,000), rabbit anti-phospho-NF-κB p65 (Cell Signaling Technology, #3033, 1:1,000), and rabbit anti-β-actin (ABclonal, AC006, 1:1,000).

Flow antibodies were procured from BioLegend: Zombie NIR Fixable Viability Kit (423106), CD11b (101212), F4/80 (123135), CD86 (105032), Gr1 (108412), Ly6C (128006), Ly6G (127626), CD3e (100306), CD4 (100408), CD8a (100752), IFN-γ (505808), GzmB (396414), CD45 (103114), CD69 (104520), and FoxP3 (320029).

### Cell culture

Lewis murine lung tumor cells, CMT167 murine lung tumor cells SCC7 murine head and neck squamous carcinoma cells, murine macrophage RAW264.7 cells, H1299 human lung carcinoma cells, and A549 human lung carcinoma cells were acquired from the American Type Culture Collection. Incubation was conducted with complete medium [supplemented with 10% fetal bovine serum (FBS), penicillin–streptomycin solution (100 U/mL)] at 37°C with 5% CO_2_. Dulbecco’s modified Eagle’s medium (DMEM) was used to maintain Lewis, CMT167, SCC7, and RAW264.7 cells, while RPMI 1640 medium was used to maintain H1299 and A549 cells. All lines were routinely tested for mycoplasma contamination, but no contamination was found.

### Transwell coculture experiment

BMDMs were collected from the femurs of 6- to 12-week-old C56BL/6 mice, followed by red blood cell (RBC) depletion and differentiation in RPMI 1640 medium supplemented with 10% FBS and MCSF (20 ng/mL, PeproTech). We seeded BMDMs in the lower chambers of 0.4-μm transwell chambers (Corning, #3421) once every 2 days with replacement medium. Irradiated Lewis cells were seeded in the upper chambers on the sixth day. The Lewis cells were irradiated with 8 Gy of 6-MV x-rays in a single dose [600 monitor unit (MU)/min, Trilogy System Linear Accelerator, Varian Medical Systems]. After coculturing for 48 h, BMDMs were harvested.

### Real-time quantitative PCR

Total RNA was extracted from cell lysates using the RNA extraction kit (Omega, R6834) and measured using Thermo’s NanoDrop ND-1000. Reverse transcription of purified RNA was performed using reverse transcription reagents (Vazyme, #R323). The RT-qPCR reaction was performed using the ChamQ SYBR qPCR Master Mix (Vazyme, Q331-02) on a Step One system. Glyceraldehyde-3-phosphate dehydrogenase (GAPDH) was used to normalize gene expression using the comparative threshold cycle method. The primers sequences are listed in Table S1.

### Western blot

Protease inhibitors and phosphatase inhibitor cocktails II and III were added to RIPA buffer prior to lysing the cells. Protein samples underwent sodium dodecyl sulfate–polyacrylamide gel electrophoresis (SDS-PAGE) separation and subsequent transfer onto polyvinylidene difluoride (PVDF) membranes. Following this, the membranes were blocked at room temperature for 1 h using a solution of 5% skim milk powder in tris-buffered saline containing 0.1% Tween 20 (TBST). Subsequently, the membranes were incubated overnight at 4°C with the appropriate primary antibodies. After thorough washing and incubation with diluted secondary antibodies, the membranes were exposed to NcmECL Ultra (P10100, NCM Biotech) for imaging.

### Enzyme-linked immunosorbent assay

Analysis of MCSF of cell supernatant was done by ELISA kit (Cloud-clone, SEA090Mu, SEA090Hu).

### Chromatin immunoprecipitation

Tumor cells subjected to treatment were cross-linked with 1% formaldehyde at room temperature for 10 min. Subsequently, the cells were washed with PBS and processed according to the manufacturer’s instructions using the ChIP Assay Kit (Beyotime, P2078). Anti-NF-κB p65 (Cell Signaling Technology, #8242, 1:100) was used for immunoprecipitation. The specific primers are listed in Table S1.

### Cytokine measurement

Cytokines (MCSF, IL-4, IL-6, IL-10, TGF-β, and TSLP) in the tumor tissue were measured by LEGENDplex Custom Mouse Panel.

### Single-cell transcriptome analysis

Mice bearing Lewis lung carcinoma subcutaneous tumors after 8-Gy irradiation at 72 h were sacrificed and immersed in 75% alcohol, and the subcutaneous tumor tissues were removed using sterilized ophthalmic scissors and forceps separation. The tissue was cut into small pieces of yellow bean grain size (around 100 mg), washed 2 times with sterile PBS buffer, and immediately placed into pre-chilled tissue preservation solution. Samples were sent to Nanjing Singleron Biotechnologies for GEXSCOPE single-cell transcriptome analysis and performed data processing and analysis.

### Dual luciferase reporter assay

We constructed the wild-type and mutant plasmid of CSF1 according to the predicted binding sites between CSF1 and p65. The sequence of predicted binding site in the gene promoter is 5′→3′1944-1953 GGGGATTTTC. The sequence of mutated binding site is GCCCCT----A. The reporter gene plasmid was constructed by inserting the CSF1 promoter sequence and firefly luciferase into the pGL3-basic plasmid. The internal reference plasmid was constructed by inserting renilla luciferase into the pRL-TK plasmid. The reporter gene firefly luciferase plasmid pGL3-CSF1, the renilla luciferase plasmid pRL-TK, and the transcription factor plasmid pcDNA3.1-RELA were cotransfected into HEK-293T cells. Then, HEK-293T cells were subjected to various experimental treatments. After the treatment, the activities of the firefly luciferase and renilla luciferase were detected using Microplate Reader and the ratio of them was calculated.

### Animal experiments

Male C57BL/6 mice (6 to 8 weeks) and male C3H mice (6 to 8 weeks) were purchased from HBCDC (Wuhan, China). All animal experiments followed approved protocols by the Hubei Provincial Animal Care and Use Committee and adhered to the experimental guidelines of the Animal Experimentation Ethics Committee at Huazhong University of Science and Technology [ethical approval number: [2021] Institutional Animal Care and Use Committee (IACUC Number): 3543].

To induce subcutaneous tumors, mice were anesthetized with 1% pentobarbital sodium before all procedures. Subsequently, each mouse was subcutaneously inoculated with 750,000 Lewis cells. When tumors reached a volume of 75 mm^3^, the mice were randomly assigned to 4 groups and subjected to various treatments. Mice in the RT group underwent 3 local irradiations of 8 Gy. For PLX3397 treatment, PLX3397 preparation and dosing for xenograft experiments were performed as described [44]. PLX3397 was administered by gastric gavage at 40 mg/kg day on the same day irradiation treatment started [44]. Vernier calipers were used to measure tumor size every 2 days by a blinded reader. The tumor volumes were calculated using the following formula: *V* = (*L* × *W*^2^)/2, where *V* represents volume (mm^3^), *L* denotes length (mm), and *W* stands for width (mm).

### In vivo antitumor immunity

To analyze changes in T cells and myeloid cells within the TME, tumor tissues were collected and digested. After preparing the single-cell suspension, the cells were labeled with the Zombie NIR Fixable Viability Kit, CD45, CD3, CD4, CD8, CD11b, F4/80, Gr1, Ly6C, and Ly6G and then incubated at 4°C for 30 min. For intracellular staining of IFN-γ, GzmB, and FoxP3 in T lymphocytes, cells were fixed and permeabilized after stimulation with monensin sodium salt, Na^+^ ionophore, and phorbol 12-myristate 13-acetate for 5 h at 37°C with 5% CO_2_.

To analyze the alterations in T cells and myeloid cells within the spleens of mice, the spleens were minced and centrifuged at 500*g* for 5 min to isolate the cells. After preparing the single-cell suspension, the cells were stained with anti-mouse Zombie NIR Fixable Viability Kit, CD45, CD3, CD4, CD8, CD69, CD11b, F4/80, Gr1, Ly6G, and Ly6C and incubated at 4°C for 30 min.

### Macrophage depletion

For the macrophage depletion study, intraperitoneal injections of 200 μl of Clo (FormuMax, F70101C-AC) were administered every 3 days, commencing the day before treatment

### Blood routine examination of patients

A retrospective analysis was performed to examine alterations in monocyte proportions in the peripheral blood of 30 non-small cell lung cancer patients before and after RT, who had not received chemotherapy. This study received approval from the Ethics Committee of Union Hospital, Tongji Medical College, Huazhong University of Science and Technology (ethical approval number: UHCT-IEC-SOP-016-03-01).

### Statistical analysis

Statistical analyses were conducted using Prism software (GraphPad Prism 6.0). All in vitro experiments were independently performed in triplicate. Group comparisons were evaluated using Student’s *t* test (2-tailed) and one-way analysis of variance (ANOVA) with Tukey’s multiple comparisons test. Quantitative data are expressed as mean ± SEM. Statistical significance was set at a threshold of *P* < 0.05, denoted as * for *P* < 0.05, ** for *P* < 0.01, *** for *P* < 0.001, and NS for not significant.
