# Supplementary material for: Targeting the Cascade Amplification of Macrophage Colony-stimulating Factor to Alleviate the Immunosuppressive Effects Following Radiotherapy
Source: Research (Wash D C). 2024 Aug 20;7:0450. doi: 10.34133/research.0450 (PMC11334716; doi:10.34133/research.0450)
Supplement: Supplementary 1 — Figs. S1 to S11 Table S1 [file research.0450.f1.docx]

**Supplemental Material**

**
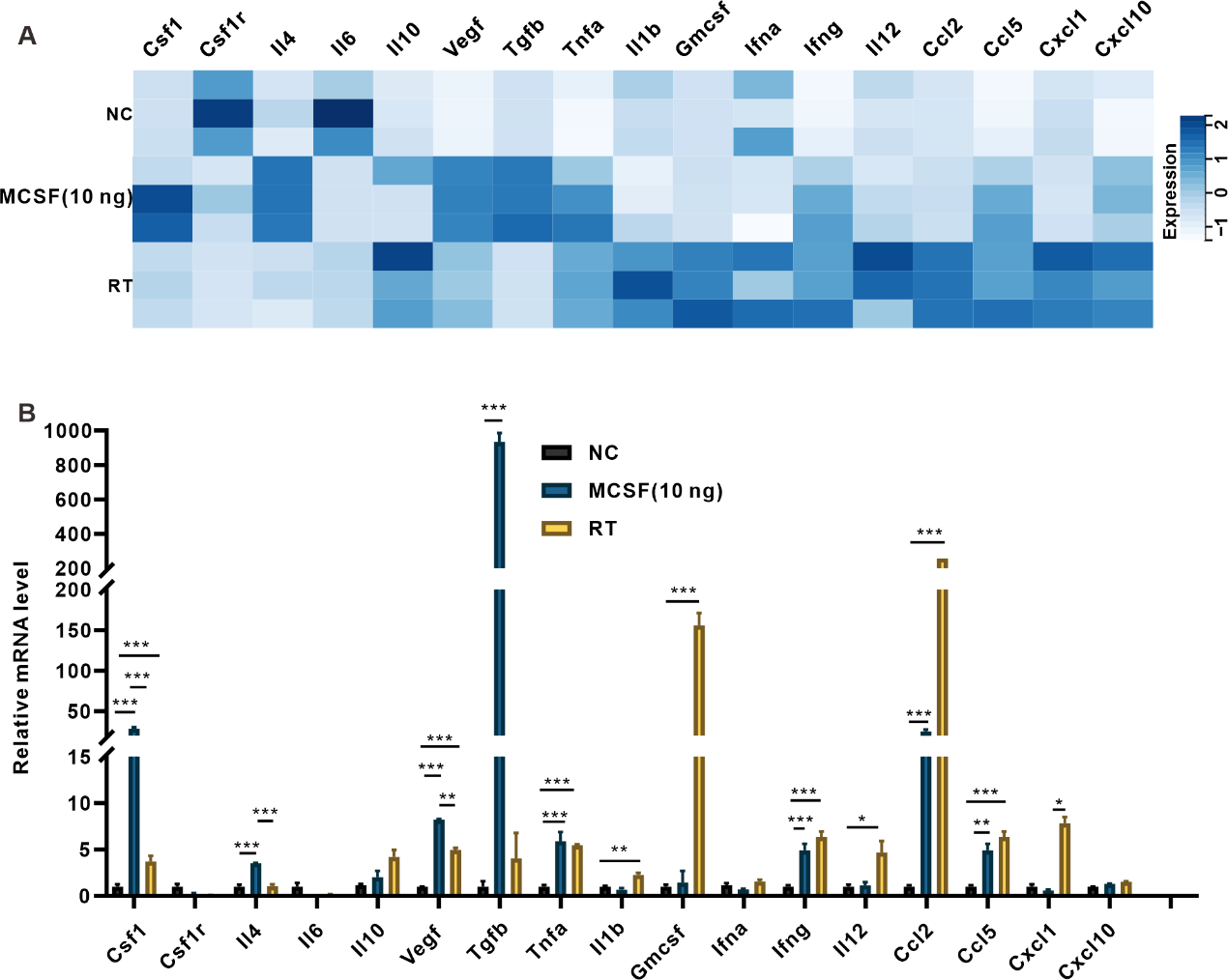
**

**Fig. S1 MCSF supplementation affects transcription levels of multiple cytokines.**

(A) In Lewis cells treated with MCSF (10ng) and subjected to irradiation, RT-qPCR was used to assess the expression levels of cytokines. (B) Statistical plot of differential cytokine mRNA level in Lewis cells.


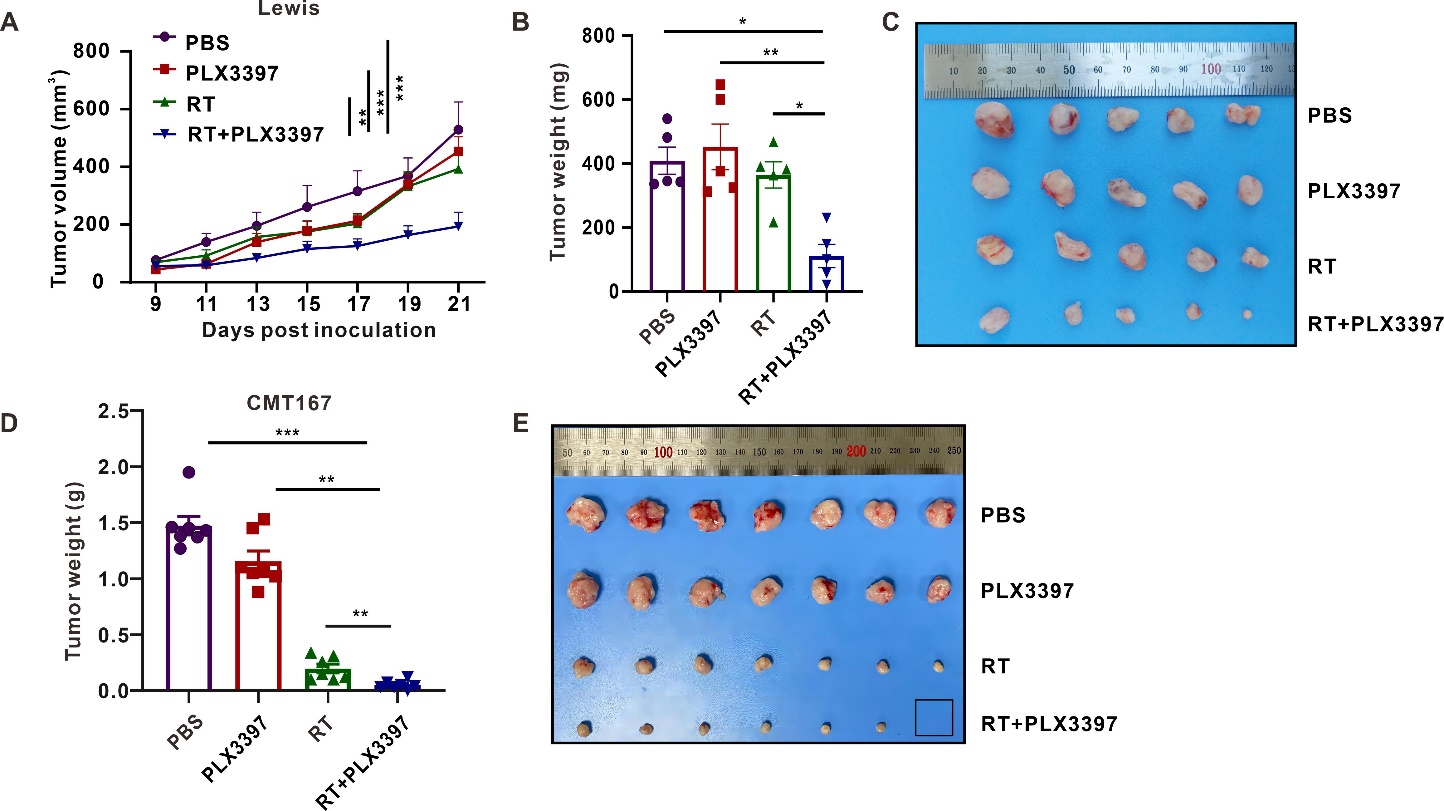


**Fig. S2 PLX3397 treatment after irradiation inhibits tumor growth.**

(A) Tumor growth curves of the Lewis subcutaneous transplant tumor model subjected to different treatment (n = 5 per group). (B) Tumor weights were measured in the Lewis subcutaneous transplant tumor model after different treatments (n = 5 per group). (C) A visual representation of the tumors in Lewis subcutaneous transplant tumor models in different groups. (D) Tumor weights were measured in the CMT167 subcutaneous transplant tumor model after different treatments (n = 7 per group). (E) A visual representation of the tumors in CMT167 subcutaneous transplant tumor models in different groups.

**
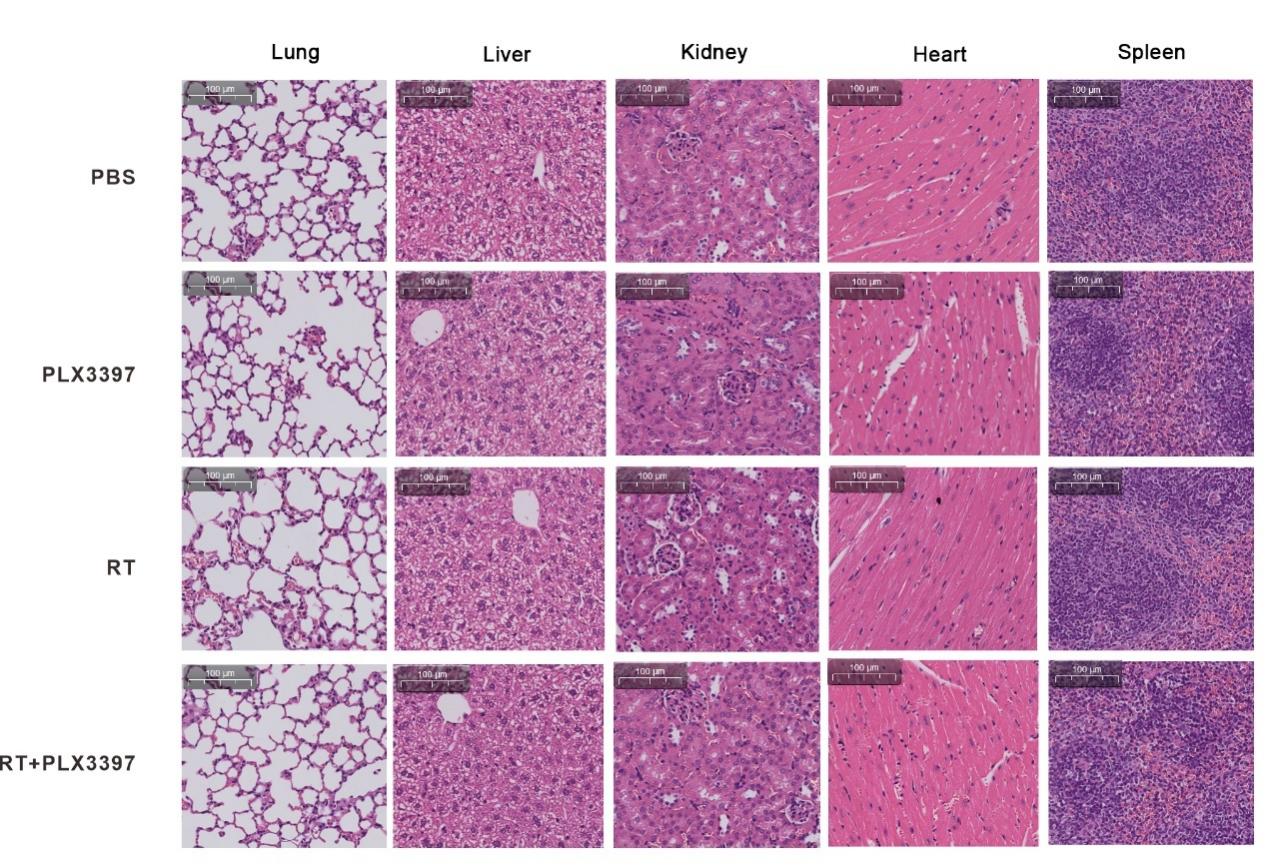
**

**Fig. S3 Combination of RT and PLX3397 demonstrates good biocompatibility.**

Examining the main organs of mice from different groups histologically with hematoxylin and eosin. Scale bars, 100 μm.

**
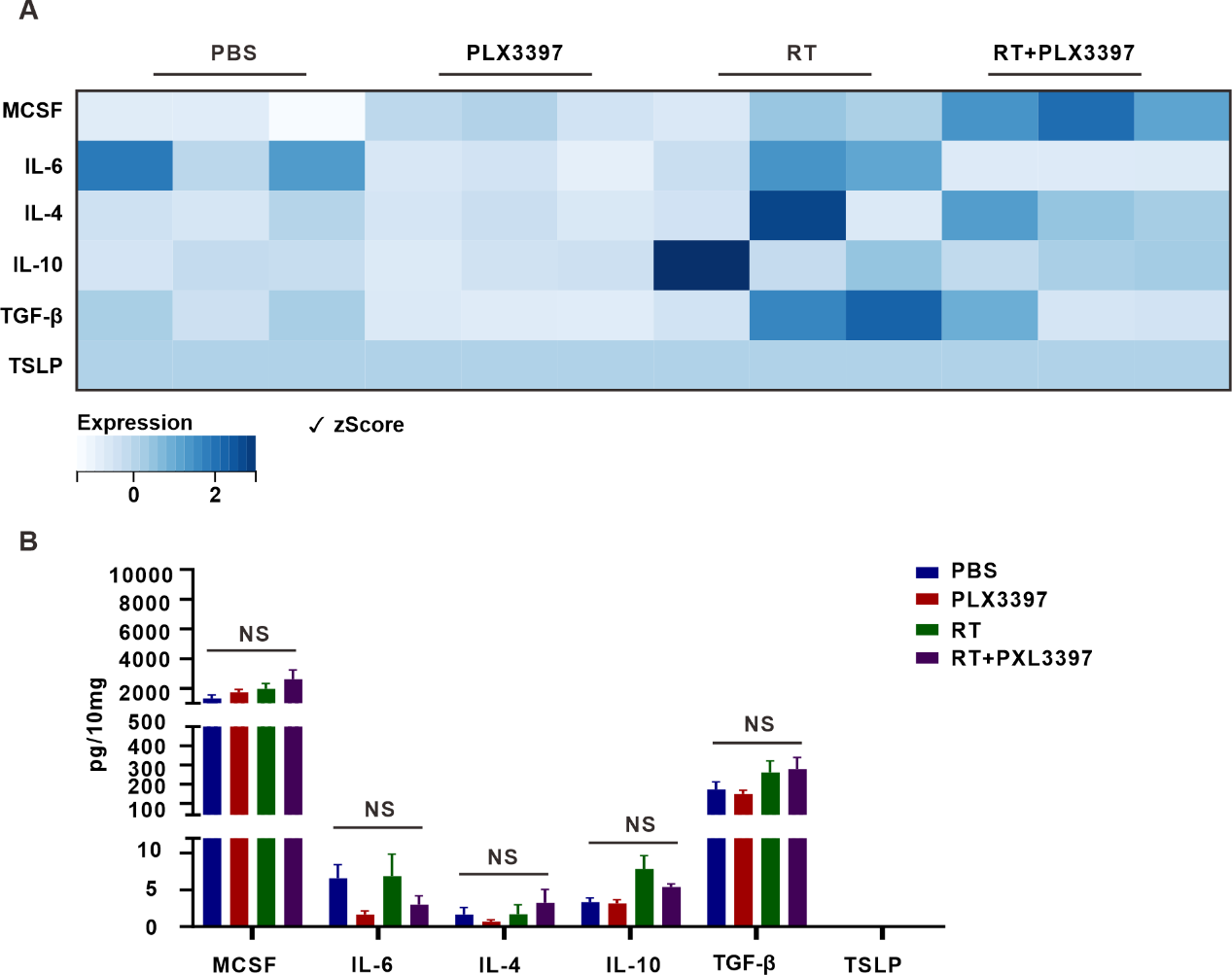
**

**Fig. S4 Blocking MCSF pathway does not affect local inhibitory cytokines secretion.**

(Α) Heatmap of differential cytokine expression in Lewis subcutaneous tumors in corresponding treatment groups. (B) Statistical plot of differential cytokine expression in subcutaneous tumors.


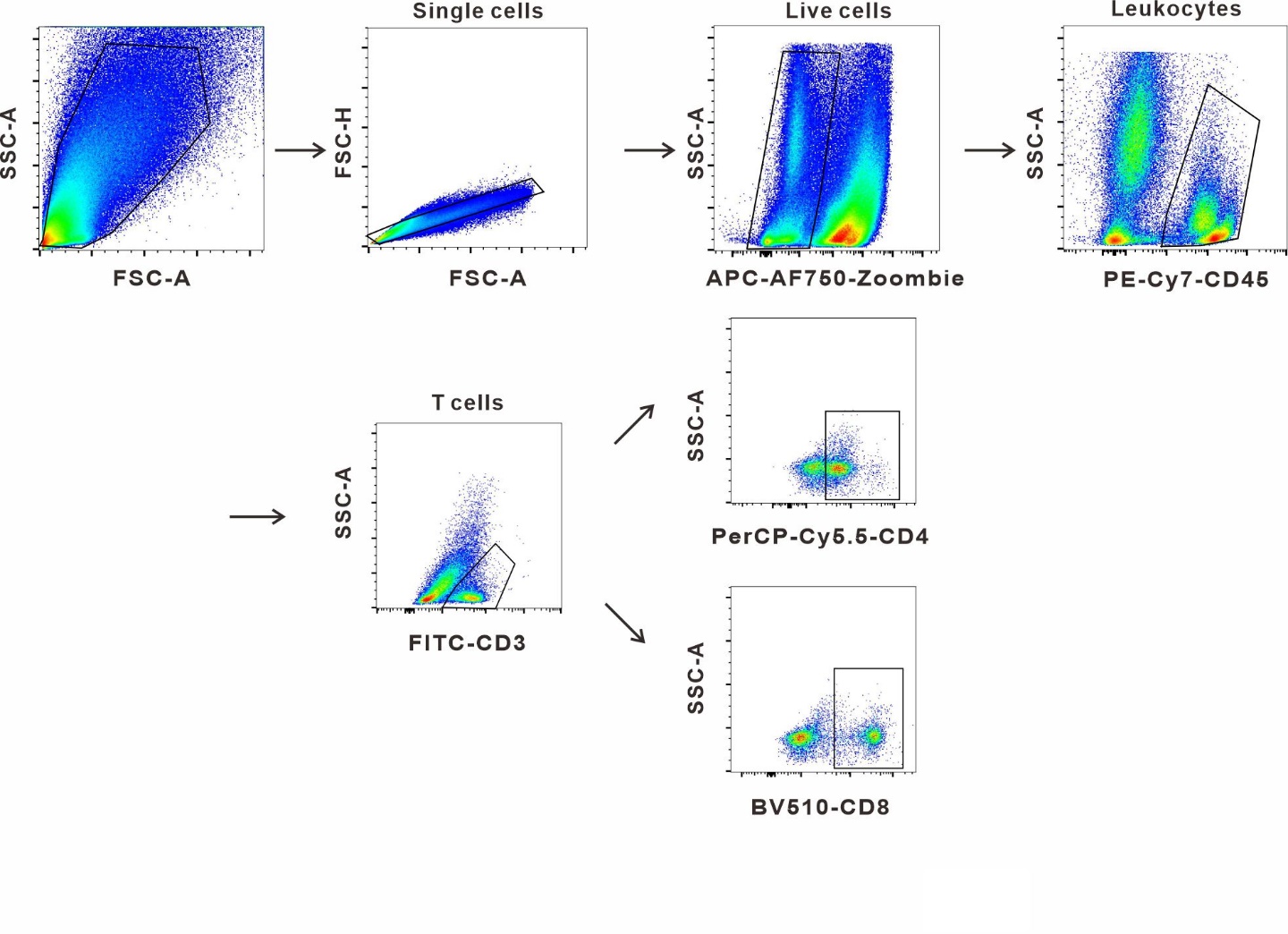


**Fig. S5 Flow cytometry gating strategy for the measurement of T cells.**


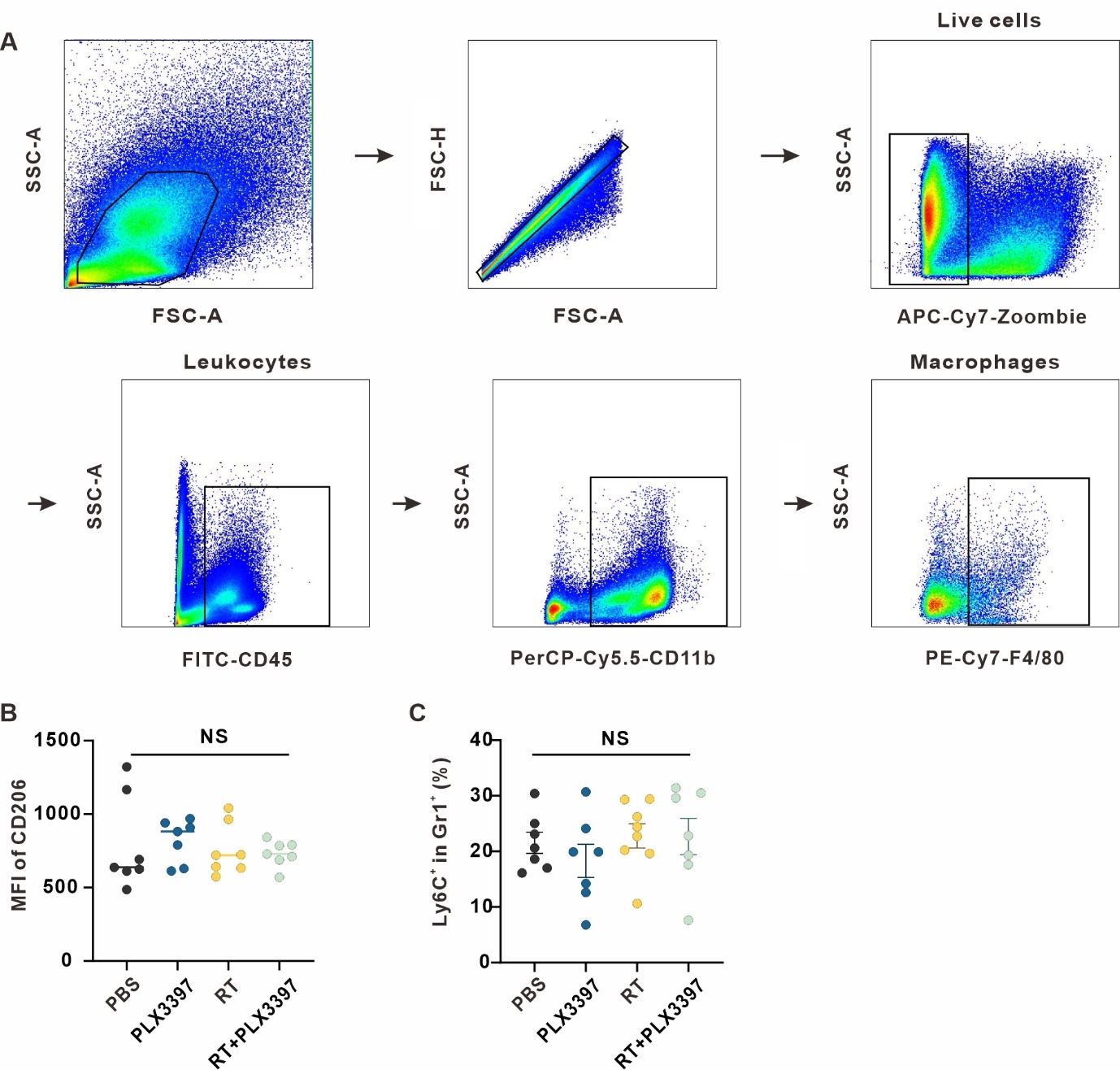


**Fig.** **S6 Flow cytometry gating strategy for the measurement of myeloid cells.**

(A) Flow cytometry gating strategy was employed to measure myeloid cells. (B) Alterations in the expression of CD206 within CD11b^+^F4/80^+^ cells were analyzed in Lewis subcutaneous tumors across the respective treatment groups (n= 7 per group). (C) Alterations in the proportion of Ly6C^+^ cells within Gr1^+^ cells were analyzed in SCC7 subcutaneous tumors across the respective treatment groups (n= 7 per group).

**
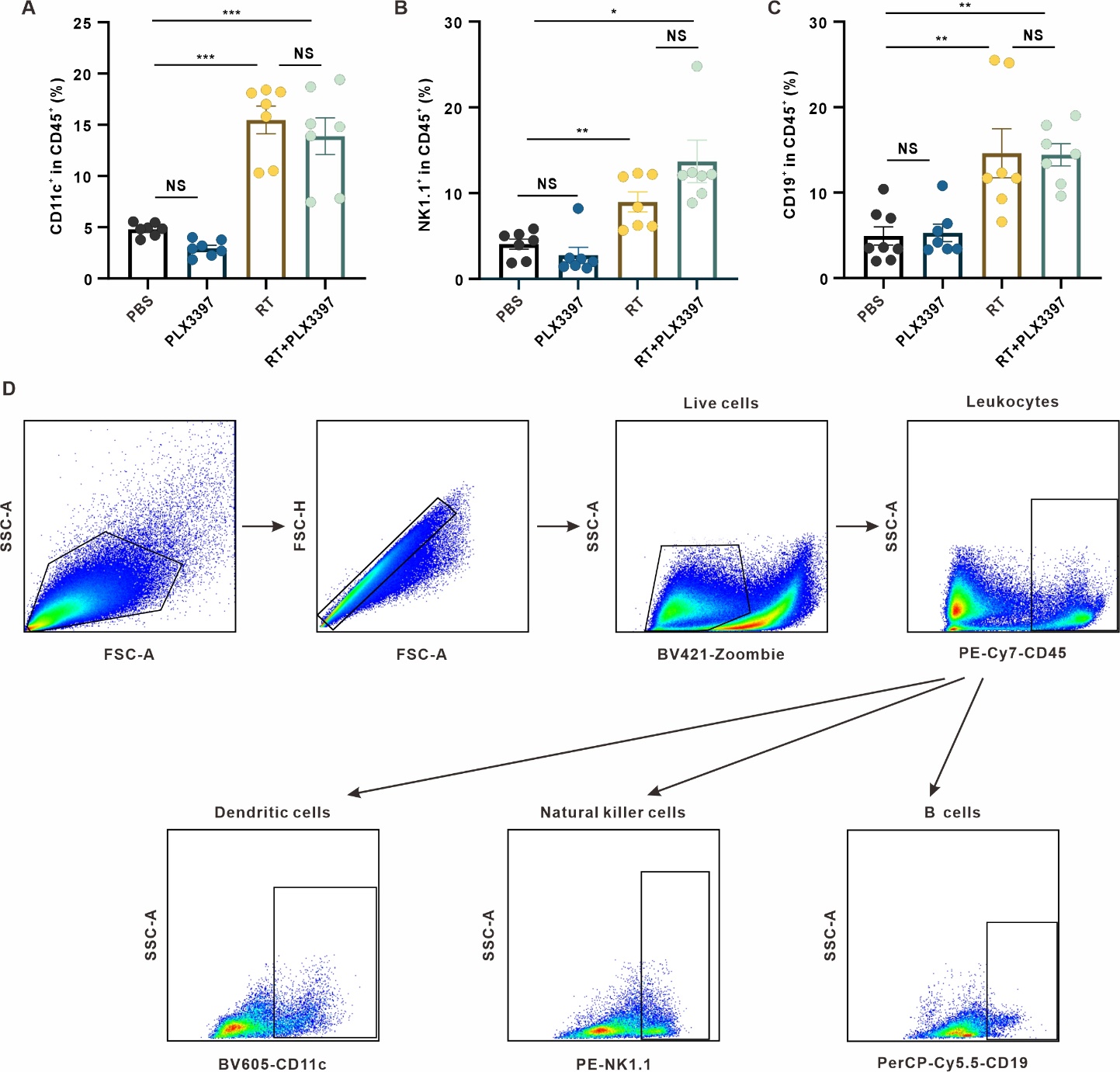
**

**Fig. S7 Flow cytometry analysis of DCs, NK cells and B cells.**

(A-C) Flow cytometry analysis presenting variations in DC, NK, and B cell populations within the tumor of the Lewis subcutaneous transplant tumor model in response to the designated treatment regimens (n = 7 per group). (D) Flow cytometry gating strategy was employed to measure DC, NK, and B cell populations.

**
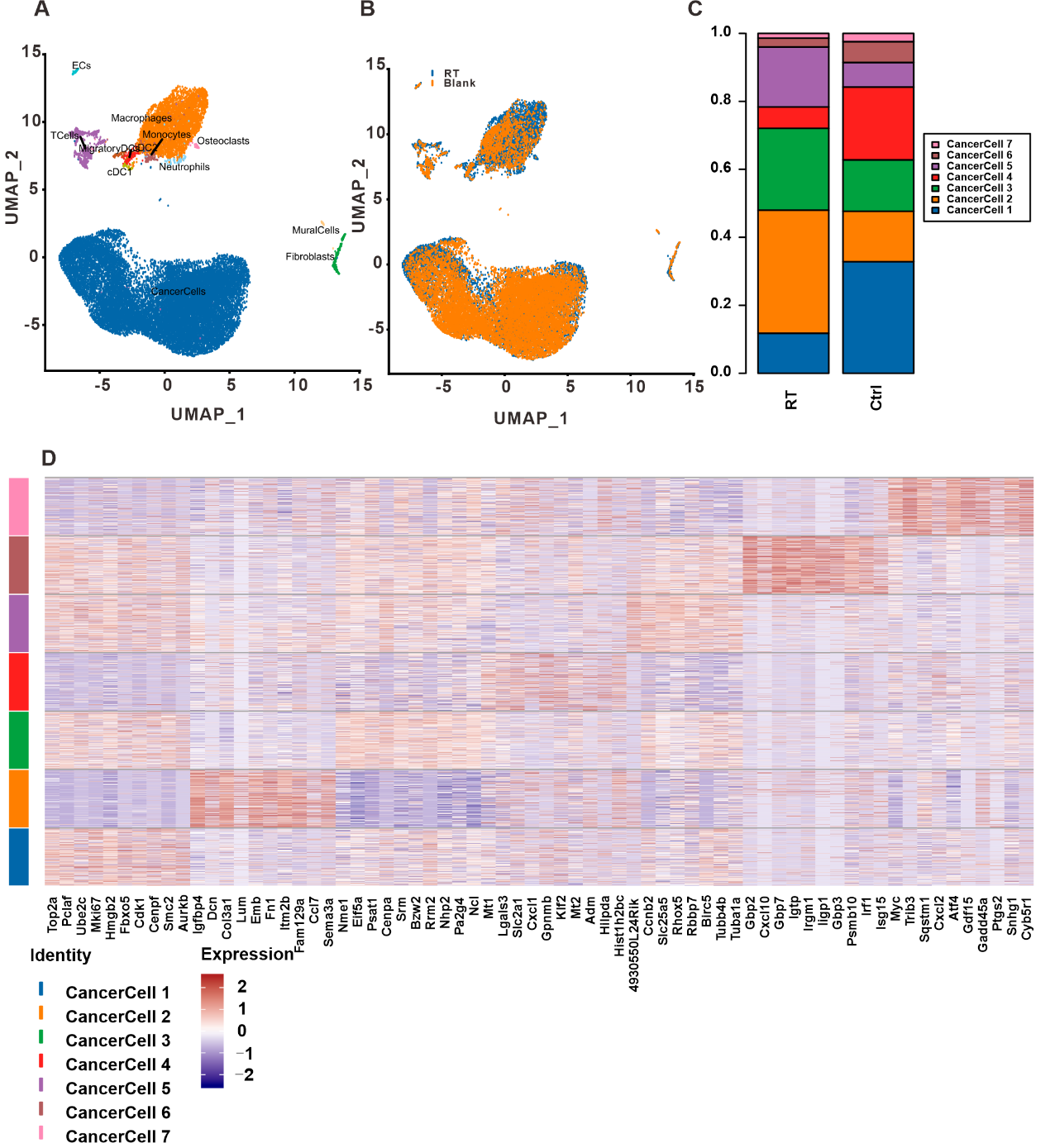
**

**Fig.** **S8 Single cell sequencing in unirradiated and irradiated Lewis subcutaneous tumors.**

(A-B) UMAP representation for unirradiated (Ctrl) and irradiated (RT) tumor tissue. Color-coded for cell type. (C) Histograms of single sample cell compositions. (D) Heatmap of differential gene expression in cancer cell subsets.

**
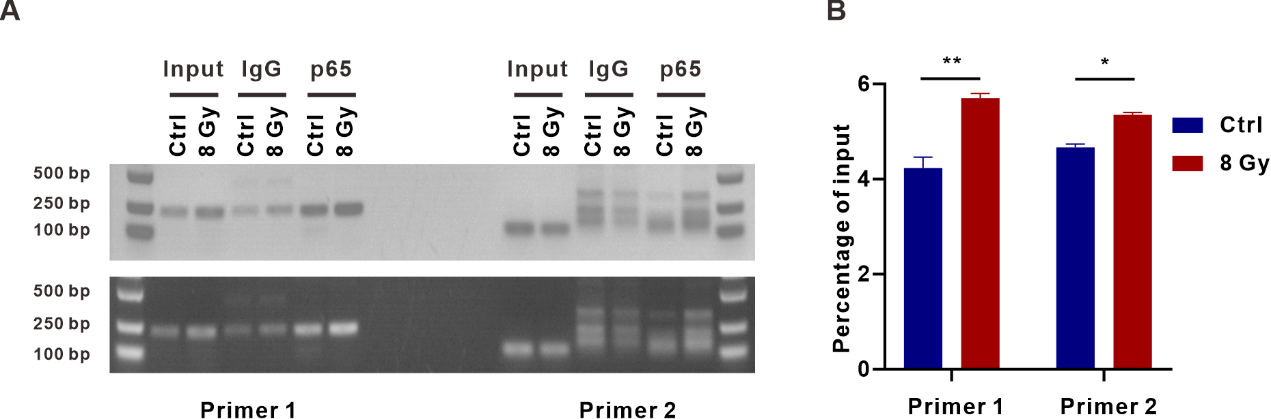
**

**Fig. S9 P65 binding to the MCSF gene promoter is enhanced after irradiation.**

1. Representative images of gel electrophoresis. (B) ChIP assay was performed in unirradiated

and irradiated Lewis cells.


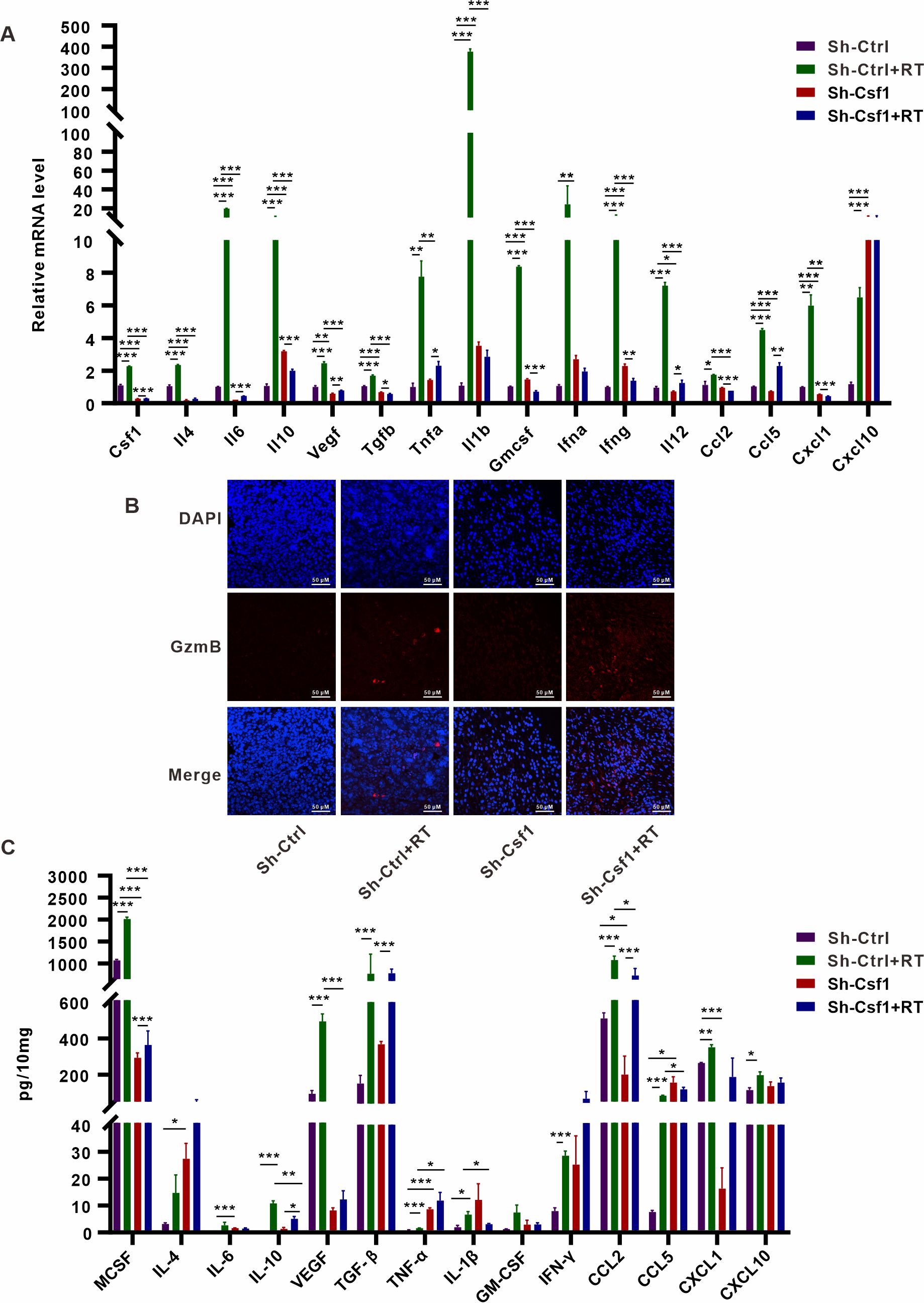


**Fig. S10** **Reducing tumor derived MCSF regulates the cytokine cascade amplification effect after irradiation.**

1. Statistical plot of differential cytokine mRNA level in Lewis cells. (B) Immunofluorescence

staining of GzmB (red) expression on the tumor tissue after different treatments. (C) Statistical plot of differential cytokine mRNA level in Lewis subcutaneous tumors.


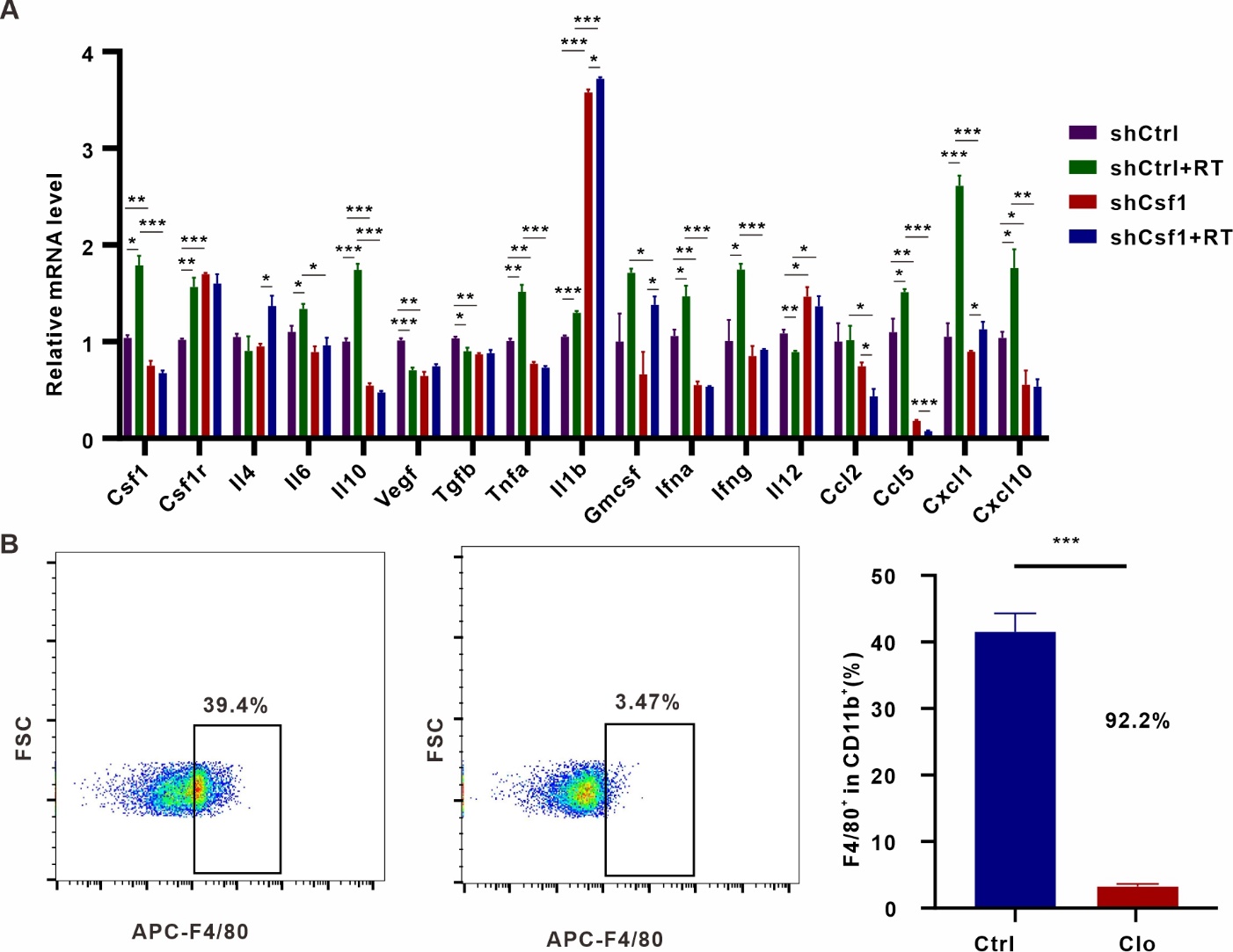


**Fig. S11 MCSF secreted by tumor cells after irradiation alters the cytokine profile of macrophages.**

1. Statistical plot of differential cytokine expression in macrophages co-cultured with irradiated

Lewis cells. (B) Flow cytometry analysis of clearance efficiency of Clo on macrophages in the spleen.

| Genes | Sequences |
| --- | --- |
| GAPDH | GAAGCTGTTGCAGCCTAGTC (forward)  CCATGGAGAAGGCCGGGG (reverse) |
| CSF1 | AACAGCTTTGCTAAGTGCTCTA (forward)  ACTTCCACTTGTAGAACAGGAG (reverse) |
| CSF1R | CGAAGTGGGATTCAACGTTATC (forward)  CCACACAAGAATATATGCCAGC (reverse) |
| TNF-A | ATGTCTCAGCCTCTTCTCATTC (forward)  GCTTGTCACTCGAATTTTGAGA (reverse) |
| IL-1B | ACCCCAAAAGATGAAGGGCTG (forward)  TACTGCCTGCCTGAAGCTCT (reverse) |
| IFN-G | GAGCCAGATTATCTCTTTCTACCT (forward)  GTTGTTGACCTCAAACTTGGC (reverse) |
| IFN-A | TGATGGTCTTGGTGGTGAT (forward)  TTGTGCCAGGAGTGTCAA (reverse) |
| IFN-B | GGTGGAATGAGACTATTGTTG (forward)  GGTGGAATGAGACTATTGTTG (reverse) |
| TGFB1 | AACAATTCCTGGCGTTACCT (forward)  GGCTGATCCCGTTGATTTCC (reverse) |
| IL-4 | GGTCTCAACCCCCAGCTAGT (forward)  GCCGATGATCTCTCTCAAGTGAT (reverse) |
| IL-6 | GAGGATACCACTCCCAACAGACC (forward)  AAGTGCATCATCGTTGTTCATACA (reverse) |
| CCL2 | TTAAAAACCTGGATCGGAACCAA (forward)  GCATTAGCTTCAGATTTACGGGT (reverse) |
| CCL5 | GACACCACTCCCTGCTGCTTTG (forward)  CTCTGGGTTGGCACACACTTGG (reverse) |
| CXCL1 | ACTGCACCCAAACCGAAGTC (forward)  TGGGGACACCTTTTAGCATCTT (reverse) |
| CXCL10 | AGTCTGAGTGGGACTCAAGGGA (forward)  CAAGCTTCCCTATGGCCCTCAT (reverse) |
| GM-CSF | TTCAAGAAGCTAACATGTGTGC (forward)  GGTAACTTGTGTTTCACAGTCC (reverse) |
| VEGF | GGTAACTTGTGTTTCACAGTCC (forward)  TTTCTCCGCTCTGAACAAGG (reverse) |
| TSLP | ACTGCAACTTCACGTCAATTAC (forward)  CGAACTTAGCCCCTTTCAAATC (reverse) |

**Table S1. Sequences of primers.**
